# The impact of HCN4 channels on CNS brain networks as a new target in pain development

**DOI:** 10.3389/fnetp.2023.1090502

**Published:** 2023-07-10

**Authors:** Maximilian Häfele, Silke Kreitz, Andreas Ludwig, Andreas Hess, Isabel Wank

**Affiliations:** ^1^ Institute of Experimental and Clinical Pharmacology and Toxicology, Friedrich-Alexander-Universität Erlangen-Nürnberg, Erlangen, Germany; ^2^ Institute of Neuroradiology, University Hospital Erlangen, Friedrich-Alexander-Universität Erlangen-Nürnberg, Erlangen, Germany; ^3^ FAU NeW—Research Center for New Bioactive Compounds, Friedrich-Alexander-Universität Erlangen-Nürnberg, Erlangen, Germany

**Keywords:** HCN4 channels, pain, BOLD-fMRI, thermal hypersensitivity, resting state-fMRI, graphtheory, multi modal MRI, knockout mice

## Abstract

While it is well established that the isoform 2 of the hyperpolarization-activated cyclic nucleotide-gated cation channel (HCN2) plays an important role in the development and maintenance of pain, the role of the closely related HCN4 isoform in the processing of nociceptive signals is not known. HCN4 channels are highly expressed in the thalamus, a region important for stimulus transmission and information processing. We used a brain-specific HCN4-knockout mouse line (HCN4-KO) to explore the role of HCN4 channels in acute nociceptive processing using several behavioral tests as well as a multimodal magnetic resonance imaging (MRI) approach. Functional MRI (fMRI) brain responses were measured during acute peripheral thermal stimulation complemented by resting state (RS) before and after stimulation. The data were analyzed by conventional and graph-theoretical approaches. Finally, high-resolution anatomical brain data were acquired. HCN4-KO animals showed a central thermal, but not a mechanical hypersensitivity in behavioral experiments. The open field analysis showed no significant differences in motor readouts between HCN4-KO and controls but uncovered increased anxiety in the HCN4-KO mice. Thermal stimulus-driven fMRI (s-fMRI) data revealed increased response volumes and response amplitudes for HCN4-KO, most pronounced at lower stimulation temperatures in the subcortical input, the amygdala as well as in limbic/hippocampal regions, and in the cerebellum. These findings could be cross-validated by graph-theoretical analyses. Assessment of short-term RS before and after thermal stimulation revealed that stimulation-related modulations of the functional connectivity only occurred in control animals. This was consistent with the finding that the hippocampus was found to be smaller in HCN4-KO. In summary, the deletion of HCN4 channels impacts on processing of acute nociception, which is remarkably manifested as a thermal hypersensitive phenotype. This was mediated by the key regions hypothalamus, somatosensory cortex, cerebellum and the amygdala. As consequence, HCN4-KO mice were more anxious, and their brain-wide RS functional connectivity could not be modulated by thermal nociceptive stimulation.

## 1 Introduction

Pain is a vital warning sign. In the recent years, HCN channels, especially HCN2, have been shown to play an important role in the development and maintenance of pain (Emery et al., 2011; Schnorr et al., 2014; Herrmann et al., 2017; Ciotu et al., 2019). HCN2 channels are critically involved in the sensitization of peripheral sensory neurons (nociceptors) in inflammatory and neuropathic pain. Nociception-related behavioral assays have shown that a global knockout of the HCN2 channel in mice results in reduced thermal hyperalgesia induced by paw injection of prostaglandin E_2_ (PGE2) (Emery et al., 2011). Mice suffering from chronic inflammation exhibited increased expression of the HCN2 channel, which in turn led to increased sensitization in pain transduction. A global HCN2-knockout on the other hand resulted in reduced thermal hypersensitivity in chronic inflammatory conditions (Schnorr et al., 2014). In contrast to that, the impact of HCN4 channels on pain development and maintenance has not been examined yet. Based on their sequence homology and basic biophysical characteristics, HCN4 channels are closely related to HCN2 (Ludwig et al., 1999; Wahl-Schott and Biel, 2009). Both channels are similar in terms of their mode of activation—both are cation channels activated by a hyperpolarized membrane potential—and in the fact that the channel activation can be influenced by cyclic adenosine monophosphate (cAMP). Beyond these similarities, however, the two channels differ significantly in other aspects. The HCN2 channels are activated much faster (τ-value between 100 and 450 ms) than the HCN4 channels with activation kinetics ranging from a few hundred milliseconds to a few seconds. Another distinct difference between the two isoforms is their expression pattern. While HCN2 is found in virtually all neurons, HCN4 is absent from peripheral neurons and the dorsal root ganglion. In the central nervous system (CNS), HCN4 is most highly expressed in the thalamus (particularly the lateral nuclear group) and in the olfactory bulb. Moderate expression of HCN4 is found in the basolateral amygdala and in deep cerebellar nuclei, and low-level expression is detected in hippocampus, cerebral cortex, and brainstem nuclei (Moosmang et al., 1999; Santoro et al., 2000; Heintz, 2004; Zobeiri et al., 2019).

The thalamus is generally responsible for stimulus transmission and is therefore essential for information processing in the CNS. In particular, areas of the ventral posterior nucleus are responsible for the transmission and processing of mechanical and thermal stimuli (including painful ones) (Bushnell and Duncan, 1987; Lenz, 1992).

Information entering the CNS is filtered and processed by the thalamus, and the processed signals are transmitted to higher brain centers. Moreover, pathophysiological conditions such as chronic pain states are reflected by irregularities in the firing rate of thalamic neurons and disturbed thalamocortical oscillations (Walton et al., 2010). It is known that the current (I_h_) carried by HCN channels including HCN4, controls the excitability of thalamocortical neurons and regulates rhythmic thalamocortical oscillations (Robinson and Siegelbaum, 2003; Zobeiri et al., 2019).

Experiments in rats demonstrated that intra-thalamic (Ding et al., 2016) and intra-cerebrovascular (Yang et al., 2019) infusions of the unselective HCN channel blocker ZD7288 were able to significantly reduce chronic pain. However, the specific HCN channel isoform(s) mediating this effect are not yet known.

The HCN2 channel, which is related to the HCN4 channel, has already been shown to play a role in the development and maintenance of pain. For HCN2, it has been shown that chronic inflammation leads to HCN2 overexpression, which is accompanied by sensitization of peripheral nociceptors. In addition, knockout of HCN2 channels resulted in thermal hyperalgesia in mice. Since both channel isoforms - HCN2 and HCN4—share some characteristics such as the pacemaker ability (Stieber et al., 2003; Wahl-Schott and Biel, 2009; He et al., 2014), we hypothesized that HCN4 channels would have a comparable effect on nociception.

To gain further insight into this issue, we conducted an exploratory data-driven study aimed at characterizing the influence of HCN4 channels on nociception by comparing brain-specific HCN4-knockout mice (HCN4-KO) with control littermates (Ctr). First, we characterized motor activity, anxiety, nociceptive and reflexive behavior by using a battery of behavioral tests.

Second, to investigate the influence of HCN4 channels on the cerebral modulation of nociception, both, stimulus-driven fMRI (s-fMRI) and resting state MRI (RS-fMRI) were performed. A conventional analysis should provide a first impression of the brain structures involved in nociception with respect to their activated volume, signal amplitude, activation probability and time to peak evoked by the application of thermal stimuli. A graph-theoretical analysis of the data obtained was performed to gain additional insight into the communication ability of the structures identified so far.

Finally, high-resolution anatomical MRI data sets were acquired *ex vivo* to investigate the general brain anatomy and whether possible changes in HCN4-KO mice might be related to nociceptive processing.

## 2 Methods

### 2.1 Animal description and housing

**FIGURE 1:**
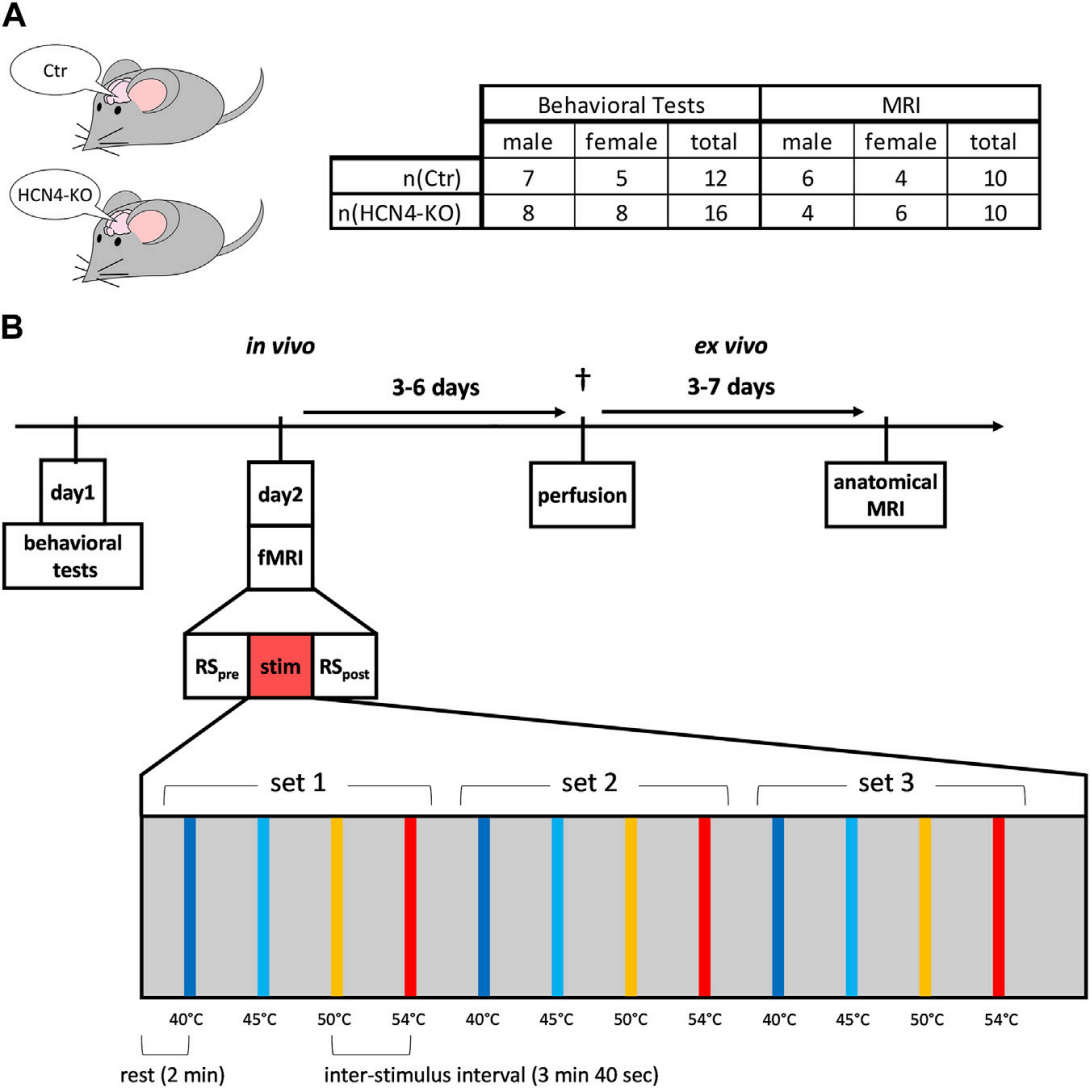
Study design. **(A)** Male and female brain-specific HCN4-knockout mice (HCN4-KO) and their littermates (Ctr) were used for this study. **(B)** After habituating the animals to the behavioral setups (except open field), the behavioral assessments were performed on day 1, followed by the functional MRI measurements consisting of an initial resting state measurement (pre RS), followed by the thermal stimulation fMRI experiment and the final resting state measurement (post RS) at the end. During thermal stimulation, three sets of sequentially increasing thermal stimuli were applied to the right hind paw, ranging from innocuous 40°C to noxious 54°C. Three to 6 days later, the animals were perfused. Within three to 7 days after the perfusion, the high-resolution anatomical MRI took place.

The mouse lines used were generated as described (Zobeiri et al., 2019). As the global knockout of HCN4 is embryonic lethal (Stieber et al., 2003), we used floxed HCN4 animals (Stieber et al., 2003) and the Nestin-Cre transgene (Tronche et al., 1999) to allow for brain-specific deletion of HCN4. Brain-specific HCN4-knockouts carried the genotype HCN4^L2/L1^, Nestin-Cre^tg/0^, controls were litter-matched HCN4^L2/+^, Nestin-Cre^0/0^. L2 denotes the floxed allele, L1 indicates the floxed-out locus. Mice were housed in groups of maximal five animals at 21°C in a 12 h-light/dark cycle. Food (Ssniff, Soest, Germany) and water were provided *ad libitum*. Male and female mice were used for the experiments. Data from both sexes were pooled for all analyses. Mice were at least 12 weeks old to ensure that brain development was largely complete (Hammelrath et al., 2016). Animal numbers for the behavioral tests were 7 males and 5 females for Ctr, 8 males and 8 females for HCN4-KO. For MRI, 6 males and 4 females were used for Ctr, 4 males and 6 females for HCN4-KO (Figure 1A). The number of animals between the behavioral tests and the fMRI experiment differed because the behavioral tests also included animals intended for a different set of experiments. However, the experimental design was the same until the end of the behavioral testing.

The study was conducted in accordance with Directive 2010/63/EU of the European Parliament and approved by the local regulatory authority (Regierung von Unterfranken) under license number RUF-55.2.2-2532-2-1460-16.

### 2.2 Study design

The influence of HCN4 channels on motor activity, anxiety, reflexive behavior, peripheral and central acute nociception, as well as brain function and anatomy was investigated in behavioral tests and by using RS-fMRI as well as s-fMRI. An additional high-resolution anatomical MRI scan allowed screening for morphological differences (Figure 1B).

At day one, the behavioral studies started with the open field test (motor behavior and anxiety), followed by the von Frey test for mechanical sensitivity and the Hargreaves test for thermal sensitivity. The tail flick test was performed to assess spinal reflexive behavior. One day later, the fMRI measurement was performed. The anatomical MRI scans were performed *ex vivo* after completion of the series.

### 2.3 Behavioral tests

The experiments were conducted during the light cycle. Mice received 4 days of training to habituate to the test environment of the withdrawal tests. Immediately prior to testing, the mice were again habituated to the test environment until the animals settled. Mice were not habituated to the open field test arena.

#### 2.3.1 Open field test

Each mouse was placed in the center of a 50 cm × 50 cm open field arena with opaque walls. The mice were then videotaped while freely exploring the open field arena for 10 min. The test was conducted under undisturbed conditions in a soundproof chamber with lights on. Anxiety was measured as the ratio of time spent in safe areas (corners and edges) as compared to open areas (interior and center). After each trial, the open field arena was disinfected to eliminate odors.

#### 2.3.2 von Frey test

The mice were placed in individual 10 cm × 10 cm × 14 cm compartments located on a perforated metal sheet with a mesh size of 5 mm × 5 mm. An electronic dynamic Plantaraesthesiometer (model 37450, Ugo Basile, Varese, Italy) was used. The 0.5 mm diameter filament was placed beneath the hind paw while the applied force was increased at 1 g/s until the mouse withdrew the paw. The reaction time and the force intensity at the withdrawal threshold were automatically recorded. Maximum force was reached after 10 s if the mouse did not withdraw. The mice were tested four times on both hind paws with an interval of 5 min.

#### 2.3.3 Hargreaves test

The mice were placed in individual 10 cm × 10 cm × 14 cm compartments, which were placed on a glass floor. The test was performed using a Hargreaves apparatus (model 37370, Ugo Basile, Varese Italy). An infrared light beam was focused on the plantar side of the hind paw. The paw withdrawal latency was automatically detected and measured four times on both hind paws with an interval of 5 min.

#### 2.3.4 Tail flick test

A modified tail flick test was performed in the same test environment and concurrently with the Hargreaves test. In contrast to the classical tail flick test, the animal remained unrestrained, thereby reducing stress and providing more reliable results. Prior to placing the mice in the chambers, the tail was marked at 1/3 of its length starting from the tail tip. The infrared light beam was focused below the marked position on the tail. The tail withdrawal latency was automatically detected and measured four times at 5-min intervals.

### 2.4 Functional MRI

All MRI measurements were acquired on a 4.7T BRUKER BioSpec (Bruker BioSpin MRI GmbH, Ettlingen, Germany) using the operating software ParaVision (V. 7.0.0 Bruker BioSpin MRI GmbH, Ettlingen, Germany). To induce anesthesia, mice received 5% isoflurane in medical air for 2.5–3 min. To maintain anesthesia during measurements, the isoflurane concentration was reduced to 0.7%–1.5% depending on the respiratory rate (target: 90–120 breaths/min for optimal pCO_2_ and blood oxygenation level dependent (BOLD) contrast with minimal head movement). The mice were then placed and secured in prone position on an acrylic cradle with an integrated water heating system to prevent hypothermia. A pediatric respiratory sensor (Graseby^®^ Respiration Sensor, Smiths Medical, Inc., Minneapolis, MN, United States) was placed under the abdomen of the mice to monitor the respiratory rate. Head movement was reduced by securing the front teeth in a nose-mouth mask, which also granted a constant supply of isoflurane for anesthesia maintenance. An eye ointment (Bepanthen, Bayer Vital GmbG, Leverkusen, Germany) was applied to prevent exsiccation damage to the eyes. The right hind paw was fixed with the dorsal side to a computer-controlled Peltier heating element to deliver thermal stimuli. A 3 cm 4-channel array head coil (Bruker BioSpin MRI GmbH, Ettlingen, Germany) placed directly above the brain was used for signal detection and good signal-to-noise ratio. Prior to the fMRI measurements, initial scout images and typical adjustments were performed to ensure the correct positioning of the mice inside of the scanner as well as to correct local field inhomogeneities. To detect possible head movement of the mice, a fast gradient-echo Echo Planar Imaging sequence (EPI; TR = 100 ms; TE_eff_ = 25.3 ms; FOV = 15 mm * 15 mm; 1 slice; slice thickness 0.5 mm; matrix 64 * 64 voxels) with 300 repetitions was performed, which can be played as a movie. If movements of more than 1 voxel were detected, the mice were repositioned on the cradle and the positioning process was repeated. After these pre-adjustments, an axial rapid acquisition with relaxation enhancement (RARE) measurement was performed (TR = 2,669 ms; TE_eff_ = 56 ms; k-space averaging 4; RARE-Factor = 8; FOV = 15 mm * 15 mm; 22 slices; slice thickness 0.5 mm; matrix 128 * 128 voxels). This measurement was used as an anatomical template for positioning the volumes of the following functional measurements (landmark: distal end of the lateral ventricle). Next, one volume of 22 coronal slices (covering the brain from Bregma −2.06–1.42 mm) with an in-plane resolution of 0.234 mm * 0.234 mm was acquired using EPI (TR = 2,000 ms; TE_ef_ = 25 ms; FOV = 15 mm * 15 mm; slice thickness 0.5 mm; matrix 64 * 64 voxels) to check image quality. Before the s-fMRI measurement, a RS measurement was acquired. This pre resting state (pre RS) measurement lasted 10 min with 300 volumes of these 22 slices. The s-fMRI measurement with 1500 volumes of the same 22 slices was acquired in 50 min. During this measurement, three sets of ascending thermal stimuli were applied to the right hind paw using the computer-controlled Peltier heating device (developed in-house) with active feedback and without interference from the MRI scanner. This allowed the stimulation temperatures to be achieved very accurately to within ±1°C. At rest, the baseline temperature of the Peltier device was 33.5°C ± 1.3°C.

For a comprehensive characterization of nociceptive processing, a stimulus set consists of four thermal stimuli with the temperatures of 40°C, 45°C, 50°C, and 54°C. While the temperatures 40°C and 45°C are innocuous, 50°C and 54°C are perceived as noxious. This range of stimulus temperatures allows a detailed study of thermal perception as well as endogenous pain inhibition.

Starting with a 2-min rest period, each stimulus was applied for 20 s (5 s ramp, 15 s plateau), with a stimulus interval of 3 min 40 s. After the s-fMRI measurement, another RS measurement was performed with the same parameters stated above, for post resting state (post RS) (Figure 1B).

After removing the mouse from the scanner, the stimulated hind paw was treated with a cooling foam spray (Bepanthen, Bayer Vital GmbH, Leverkusen, Germany) to prevent skin irritation, and the mouse was placed in an empty cage until fully awake and thereafter transferred back to its home cage.

### 2.5 Cardiac perfusion and anatomical MRI

Within three to 6 days after the completion of all functional measurements, the mice were injected subcutaneously (s.c.) with 50 U heparin and euthanized with an overdose of CO_2_. Upon death, mice were immediately removed from the CO_2_ chamber, and fixed in supine position. The chest was opened, and the diaphragm cut to expose the heart. A cannula, prepared to limit depth of penetration, was inserted into the distal end of the left ventricle. The right atrium was then incised, and the mice were perfused for 5 min with prewarmed 37°C rinsing solution (1:10 solution of contrast agent (ProHance^®^, Bracco Imaging Deutschland GmbH, Konstanz, Germany) +0.9% saline +0.1% heparin) using a peristaltic pump (flow rate 5 ml/min; 85 mmHg). Thereafter, solution was switched to fixative [1:10 solution of ProHance in 4% paraformaldehyde/phosphate buffered saline (PBS)] for another 5 min. Heads were sectioned, fixed on plastic slides, and stored in 50 ml Falcon tubes containing a 1:100 solution of ProHance in 4% buffered paraformaldehyde.

The high-resolution anatomical measurement was performed within three to 7 days after the perfusion date. 24 h prior to anatomical MRI, the solution was exchanged with 0.9% saline, to wash out excess contrast agent. For anatomical MRI, the Falcon tubes containing the brains were fixed inside a full-body birdcage resonator used for excitation and signal detection. The brains were scanned with a 3D fast low angle shot (FLASH) sequence (TR = 40.48 ms, TE_eff_ = 8.43 ms; 26 averages, FOV = 16 mm * 20 mm * 16 mm with a resolution of 0.05 mm * 0.05 mm * 0.1 mm; matrix 320 * 400 * 160 voxels) for 14 h 58 m.

### 2.6 Data analysis and statistics

#### 2.6.1 Behavioral data

The open field videos were analyzed by manually marking the tail base on 600 consecutive frames covering 10 min. The image processing software MagnAn (BioCom GbR, Uttenreuth, Germany) was used to calculate total distance travelled in meters, activity as a percentage of total movement time, average velocity in m/s, and anxiety. For calculation of anxiety, the arena was divided by a 10 cm grid into different zones. Each zone was weighted differentially, representing how anxious or safe a mouse typically feels in there: corners (100%), edge (75%), interior (25%), and center (0%). Each frame was annotated with the respective anxiety score, and an average anxiety score was calculated per mouse for the entire duration of the trial.

Data from the left and right hind paws were pooled for von Frey and Hargreaves tests. Statistical differences between Ctr and HCN4-KO were calculated using a homoscedastic Student’s *t*-test (von Frey, Hargreaves, and tail flick tests). Statistical analysis was performed in Microsoft Excel 2016 (Microsoft, Redmont, Washington, United States).

#### 2.6.2 Stimulus-driven fMRI

The software BrainVoyager QX (V. 2.0.8; Brain Innovation BV, Maastricht, Netherlands) was used for pre-processing of all fMRI data. The first two volumes were discarded, as they contain saturation effects. Thereafter, slice scan time correction (ascending interleaved, interpolation method cubic spline), motion correction to eliminate brain movements (registration to first volume; trilinear detection and sinc interpolation), spatial (Gaussian smoothing; kernel 2 pixels) and temporal smoothing (linear and non-linear high-pass filtering, kernel 12 s FWHM, FFT 9 cycles) were applied. The coupling between stimulation and BOLD signal time courses was assessed by a voxel-wise generalized linear model (GLM) analysis to obtain first-order statistical parametric maps (SPMs). Each stimulation temperature had its own predictor for the GLM analysis, which was folded with a mouse-specific two-gamma hemodynamic response function (HRF).

Further analysis was performed again in MagnAn. To obtain information about different brain regions, we had developed an in-house mouse brain atlas (Neely et al., 2010; Hess et al., 2011): 22 slices that corresponded best to our grey-scale fMRI slices were selected from Franklin and Paxinos atlas of the mouse brain (Franklin and Paxinos, 2008). 211 brain regions, divided into left and right hemispheres, were selected. The regions were color-coded and indexed with a unique ID number. Each atlas slice was registered (translation x-y-z, scaling x-y, rotation z) to match the individual animal grey-scale fMRI slices and used to create a subject-specific label mask for further analysis.

False Discovery Rate (FDR; q = 0.05) was applied to binarize the predictor-specific SPMs and correct for multiple comparisons. For each subject and predictor, the surviving voxels were multiplied by the subject-specific label mask created in the previous step. This yielded stimulus-specific labeled activity maps containing the significantly activated voxels of the 211 brain regions per animal. Average time profiles of all significantly activated voxels per brain region were calculated by applying these label masks to the BOLD time series.

The stimulus-driven data were further analyzed in two branches: the first branch, a more classical approach, generated average time profiles per brain region and predictor containing 10 time points before, 10 during and 10 after the stimulus. These time profiles were then averaged over the three representations of the given stimulus. This allowed the comparison of general BOLD activation characteristics: average BOLD signal amplitude and time to reach the maximum signal amplitude (time to peak) as well as the total number of activated voxels. The number of activated voxels was multiplied by the voxel size (0.234 mm × 0.234 mm × 0.5 mm) to obtain the response volume in mm^3^. Additionally, the activation probability in percent was calculated (how many animals show activation per voxel per brain region).

The second branch, a graph-theoretical approach, generated brain networks, here for stimulation with 45°C and 50°C, which were analyzed regarding their network properties. First, global signal regression (Fox et al., 2009) (removal of the global mean containing the stimulus responses) was performed on the full-length time profiles of each predictor per subject. For each predictor and subject, the Pearson correlation coefficient *r* was calculated between the average time courses of all possible pairs of brain regions, yielding symmetric adjacency matrices. After transforming the data into normally distributed Fisher-z-values, average adjacency matrices were calculated per predictor and experimental group. Subsequently, the Fisher-z-values were back-transformed to Pearson’s *r*.

Since the resulting matrices contain a large number of *r*-values, the density of the networks had to be adjusted to ensure good topological comparison between different networks. The optimal density was determined by first calculating the average small world index sigma for the 50°C predictor for densities ranging from 2% to 20% in 2%-steps. After normalization to the highest small world index, the vertex of the resulting hyperbola was calculated, yielding the density of 7%. At this density, the biggest changes in the network occurs, and therefore, all networks were limited to the 7% strongest positive *r*-values.

The networks of Ctr and HCN4-KO were characterized in terms of global network properties (normalized shortest pathlength *λ*, normalized clustering coefficient *γ* and the resulting small world index *σ*) as well as node-specific parameters (average node strength, node degree, clustering coefficient, pathlength, betweenness, and hubscore).

Since the density is set equal for both groups, the average degree should only differ if the number of nodes vary between groups. Thus, differences in degree reflect the size of the network. The strength shows the sum of the correlation values of all connections per node. If the ratio between strength and degree is the same for both groups, then the strength parameter is also (only) an indicator of the network size. If the ratio is different, then higher strength values reflect higher connection strength. The proportion of interconnected neighbors is displayed by the clustering coefficient. Higher clustering coefficients also account for higher overall segregation, but higher local integration of specific nodes, especially in combination with higher average shortest paths. The average shortest pathlength shows the average minimum steps to reach every other node in the network. Higher shortest paths indicate higher segregation of information flow, lower ones indicate higher integration. The hubscore describes the distributional potential of a node based on its incoming and outgoing connections, while the betweenness shows the proportion of all possible paths within the network that pass through a node. Both hubscore and betweenness are measures to describe the importance of a node to the integrity of the network. The omission of such a node leads to severe disturbances in information flow or even complete disruption of the network. As these measures are normalized, no group differences can be expected. However, significant interactions indicate differences in brain network functionality.

#### 2.6.3 Resting state fMRI

After discarding the first two volumes, preprocessing of RS data included only slice scan time correction (ascending interleaved, interpolation method cubic spline) and motion correction (registration to first brain volume; trilinear detection and sinc interpolation). Further analyses, including FFT low-pass filtering at 0.1 Hz, were again performed using MagnAn.

Label masks to identify the different brain regions were created exactly as described for the s-fMRI data. Here, the label masks were additionally trimmed with individually drawn brain masks to restrict the analysis to voxels within the brain. Next, multi-seed region analysis (MSRA) was performed as described previously (Kreitz et al., 2018). Briefly, a 0.5 mm^2^ seed region was automatically positioned at the centroid of each brain region. The Pearson correlation coefficient *r* between the average time course of the seed and all other voxels in the brain was calculated, resulting in a 22-slice 3D correlation volume per seed region. Significant correlations were determined using FDR (q = 0.05). *R*-values were converted to Fisher-z-values, and values of all voxels belonging to the same region were averaged for the given seed and back-transformed to *r*-values. This approach was repeated for all regions of the label mask, resulting in an asymmetric connectivity matrix per animal. The optimal density (7%) was determined as described above for the stimulus-driven data. As for the stimulus-driven data, the networks were characterized in terms of global network properties and node parameters.

#### 2.6.4 Anatomical MRI

After manual brain segmentation, individual subjects were aligned in a two-step diffeomorphic registration scheme using the Advanced Normalization Tools (ANTs) (Avants et al., 2009; Badea et al., 2012): First, all brains were registered to one selected animal of the cohort. Next, a cohort-specific template was created by averaging all registered brains. In the second registration step, all brains were registered to this cohort-specific template. This two-step approach has been shown to yield the best results in previous projects (Badea et al., 2012). The diffeomorphic registration provides information about the deformation of each voxel needed to fit the template (Jacobian determinants).

Finally, the cohort-specific template was registered by ANTs to the 50 µm MRI template of the Allen Mouse Brain Atlas (ABA) (Allen Institute for Brain Science, 2011).

The transformation matrices and deformation fields obtained in the previous steps were now used in a reverse registration. A modified Allen Mouse Brain Atlas (>1,000 partially very small brain regions were combined into 102 functional regions, divided into left and right hemisphere) was reverse-registered to the cohort template for deformation-based morphometry (DBM). Brain region-specific mean Jacobian determinants derived from the registration subject to template space were used for the DBM analysis to assess morphological differences in ways of average volume change per brain region. Using voxel-based morphometry in individual subject space, absolute differences in brain volume could be calculated.

#### 2.6.5 Statistical analysis of MRI data

For the classical BOLD signal analysis, statistical differences between Ctr and HCN4-KO were assessed using two-factor ANOVA (between-factor genotype and within-factor brain region). Data show the mean of the whole brain ±standard error of the mean (SEM) for total activated volume, average response amplitude and time to peak as well as the average activation probability across all brain regions ±standard deviation (SD). For significant interactions between the factors genotype and brain region found by ANOVA, a homoscedastic *t*-test was used as a *post hoc* test between corresponding brain regions (*p* < 0.05, uncorrected).

For graph-theoretical data, differences were assessed using network-based statistics (Zalesky et al., 2010). First, a homoscedastic Student’s *t*-test was calculated for comparing: 45°C Ctr vs. HCN4-KO and 50°C Ctr vs. HCN4-KO; as well as a paired *t*-test for comparing: post RS HCN4-KO vs. pre RS HCN4-KO and post RS Ctr vs. pre RS Ctr. Both tests were restricted to *α* = 0.05. The size of the largest component (cluster of connected nodes) was determined as the number of connections contained. Next, for 1,000 permutations, single animal matrices were randomly divided into two groups and t-tests were recalculated. The number of tests that resulted in larger components than the original test was noted. A corrected *p*-value was calculated by dividing this number by the number of permutations, in this case 1,000. Only when this corrected *p*-value was <0.05 were the differences found in the original test accepted as statistically significant.

Differences in anatomical aspects, i.e., deformation-based differences in morphology, were assessed using two-factor ANOVA (between-factor genotype and within-factor brain region). DBM data are presented as the difference of the mean ± standard error (SE) of the difference.

#### 2.6.6 Graph-theoretical visualization

Adjacency matrices can be displayed as brain networks, with the brain regions of interest (ROIs) as color-coded nodes and the correlation coefficients as edges. Nodes are displayed within a transparent 3D brain surface at their approximate anatomical locations. The size of the nodes represents the node degree, i.e., the number of (significant) connections per node. Unconnected nodes are omitted. Red and blue edges color-code the results of the statistical tests, the meaning of which can be found in each figure legend. The software Amira (V. 5.4.2 Thermo Fisher Scientific Inc., Waltham, United States) was used to visualize the brain networks using custom programmed visualization modules.

#### 2.6.7 Randomization and blinding

Special care was taken to randomize the order in which the animals of the different groups were measured per day to avoid daytime effects. The analyst was not blinded to the genotype of the animal, but animals of both groups were always batch-analyzed in a standardized workflow, that does not require or allow user-specified input.

## 3 Results

### 3.1 Behavioral tests

**FIGURE 2:**
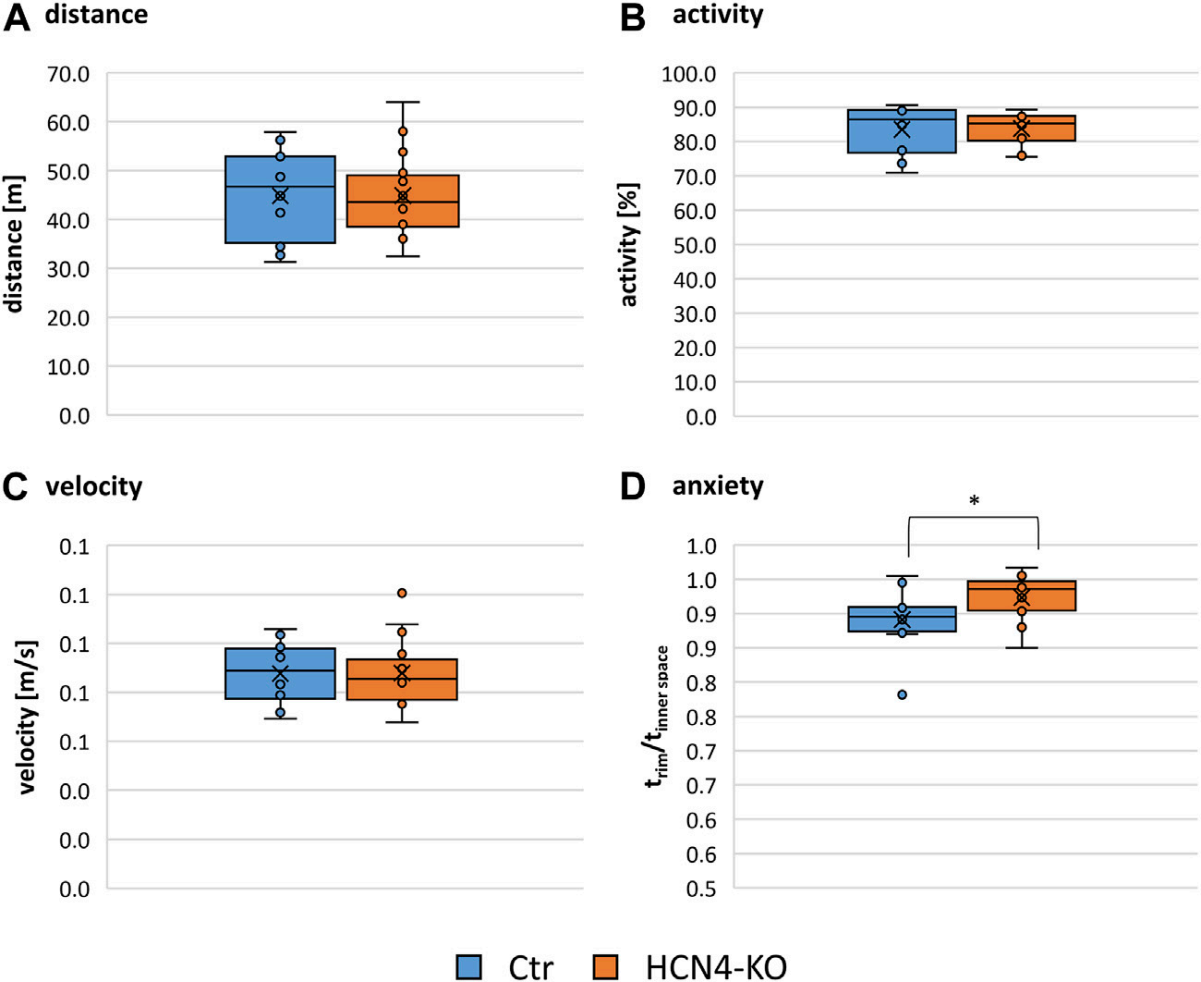
Open field results. Box plots comparing the travelled distance **(A)**, the activity as a proportion of the time spent moving compared to total time **(B)**, the average velocity **(C)** and the anxiety **(D)**, measured as a ratio of the time spent in a safe environment (corner and edge) compared to the time spent in an open environment (interior and center) of Ctr and HCN4-KO. Box spans the 25th to the 75th percentiles, the median is represented by the solid horizontal line and the mean is marked with the x. The individual values from all animals are displayed as dots. Ctr (*n* = 12) and HCN4-KO (*n* = 16); *p < 0.05 as determined by a homoscedastic *t*-test. Open field test revealed significantly increased anxiety in HCN4-KO compared to Ctr.

Differences in thermal and mechanical nociception were investigated using different nociception-related behavioral tests (Table 1; Supplementary Figure S1): The von Frey and Hargreaves test can be used to assess mechanical and thermal sensitivity as measured by paw withdrawal latencies and involves both peripheral and central mechanisms. In contrast, the tail flick test, which measures tail withdrawal latencies to thermal stimulation, is known to involve mainly spinal reflex arcs rather than central processing. The reflexive response to thermal stimulation determined by the tail flick test revealed no significant differences between HCN4-KO and Ctr. In contrast, HCN4-KO animals showed a central thermal hypersensitivity demonstrated by the Hargreaves test. Interestingly, mechanical nociception remained unaffected, in contrast to previous findings in HCN2-KO. The open field test showed no significant differences in traveled distance, activity, and average velocity between both groups (Figures 2A–C). Thus, deletion of HCN4 did not impair basic locomotion. In contrast, HCN4-KO showed significantly increased anxiety compared to controls (Figure 2D).

**TABLE 1:** Nociception-related withdrawal thresholds.

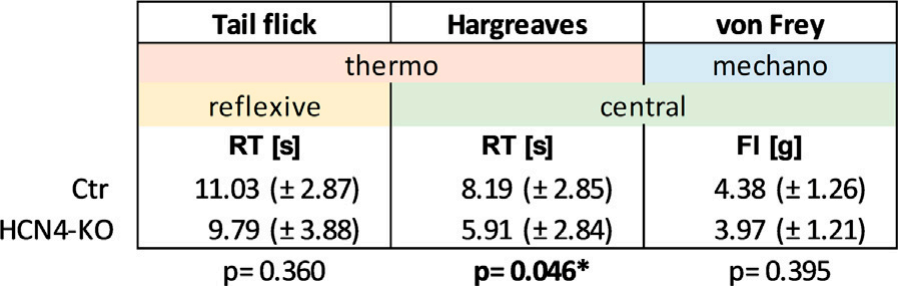

Comparison of Ctr and HCN4-KO regarding spinal reflexive tail withdrawal thresholds (tail flick, reaction time in seconds) as well as central thermal (Hargreaves, reaction time in seconds) and mechanical (von Frey test; force intensity in grams) paw withdrawal thresholds. Values are mean ± standard deviation in brackets. Ctr (*n* = 12) and HCN4-KO (*n* = 16); *p < 0.05 (bold script) as determined by a homoscedastic *t*-test. Whilst spinal reflexes were unchanged compared to Ctr, HCN4-KO in general showed a central thermal but no mechanical hypersensitivity.

### 3.2 Stimulus response analysis

FMRI data can be analyzed in different aspects: the more classical approach identifies brain regions that are significantly activated by the applied thermal stimuli and characterizes the evoked BOLD response brain-structure-wise in terms of maximum BOLD signal amplitude, time point when this maximum was reached (time to peak) and the activated brain volume. Although perhaps not a standard measure, we thought that time to peak would be informative in terms of the pacemaker ability of HCN channels in general, perhaps leading to altered temporal responses. Additionally, the activation probability can be calculated, which represents how many percent of the animals respond significantly to the stimulation.

Novel graph-theoretical approaches construct and analyze brain-wide networks based on temporal correlation of the brain-structure-specific BOLD time courses over the entire duration of the measurement, termed functional connectivity. They can be used to interpret functional relationships between brain regions as well as to characterize network functional properties with respect to information flow within the network.

First, the classical approach was used to compare BOLD signal characteristics between HCN4-KO and Ctr using two-factor ANOVAs with the factors genotype and brain region. We found significant differences in a brain-wide BOLD response analysis (Supplementary Figure S2).

**FIGURE 3:**
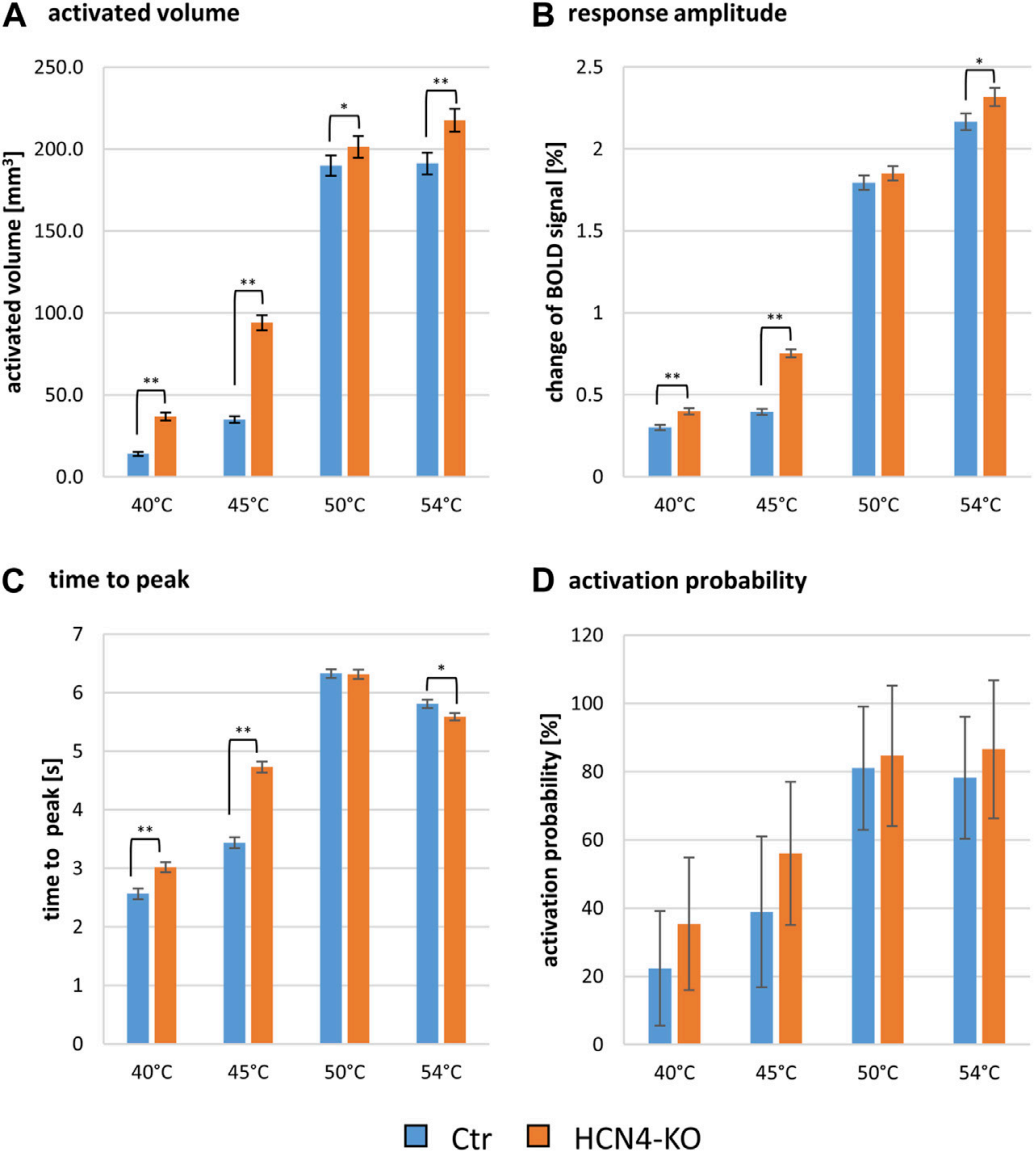
Global analysis of thermal stimulation BOLD response parameters of Ctr and HCN4-KO. **(A)** Activated brain volume in mm^3^ ± SEM, **(B)** the change of the BOLD signal in percent ±SEM, **(C)** the time from start of stimulation until the peak of the BOLD signal amplitude was reached ±SEM and **(D)** the activation probability (i.e., average number of animals responding to stimulation) ± SD at the temperatures 40°C, 45°C, 50°C, and 54°C; *p < 0.05, **p < 0.01, ***p < 0.001 were determined by a two-factor ANOVA with one within-factor containing the 211 brain regions and one between-factor genotype (Ctr, *n* = 10 and HCN4-KO, *n* = 10). For HCN4-KO, global brain-wide response analysis showed significantly larger activated volumes and response amplitudes, slower time to peak values at low temperatures and faster time to peak values at 54°C, and overall higher but insignificant response probabilities compared to Ctr.

In terms of the activated brain volume, HCN4-KO showed significantly larger activated brain volume at all four stimulation temperatures, with the most prominent changes found at lower temperatures (40°C and 45°C) (Figure 3A). ANOVA revealed an interaction between HCN4-KO and Ctr for the BOLD volume at 45°C, but not for any of the other temperatures. Therefore, a homoscedastic *t*-test was used as *post hoc* test to identify the individual brain regions in which the activated brain volume differed significantly between HCN4-KO and Ctr. In all identified brain regions (brainstem, sensory input, sensory cortex, limbic system, amygdala, hypothalamus, and cerebellum, as well as part of the anterior thalamus and dorsal hippocampus), HCN4-KO showed significantly larger activated brain volume (Table 2).

**TABLE 2:** Significant differences in activated brain volume for specific brain regions at 45°C between HCN4-KO and Ctr.

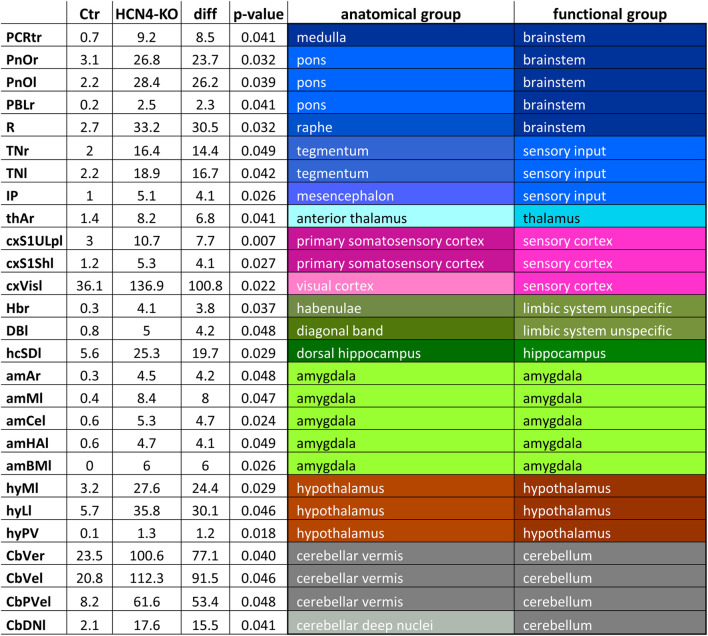

A two-factor ANOVA revealed significant interaction between the within-factor brain region and the between-factor genotype [Ctr (*n* = 10) and HCN4-KO (*n* = 10)]. Homoscedastic *t*-test was used as *post hoc* test for brain regions (*p* < 0.05; uncorrected). Brain regions with significantly larger activation volumes were in particular brainstem, sensory cortex, amygdala, hypothalamus, and cerebellum. Abbreviations: l, left; r, right; PCRtr, parvocellular reticular nucleus; PnOr, PnOl, pontine reticular nucleus oral right and left; PBLr, lateral parabrachial nucleus; R, raphe nucleus; TNr, TNl, tegmental nuclei right and left; IP, interpeduncular nucleus; thAr, anterior thalamic group; cxS1ULpl, primary somatosensory cortex upper lip; cxS1Shl, primary somatosensory cortex shoulder; cxVisl, visual cortex; HBr, habenuli; DBl, nuclei of diagonal band; hcSDl, dorsal subiculum; amAr, anterior amygdala; amMl, medial amygdaloid nucleus; amCel, central nucleus of the amygdala; amHAl, amygdala hip area; amBMl, basomedial amygdaloid nucleus; hyMl, medial hypothalamus; hyLl, lateral hypothalamus; hyPv, periventricular hypothalamic nucleus; CbVer, CbVel, cerebellar vermis right and left; CbPVel, cerebellar paravermis left; CbDNl, cerebellar deep nuclei left.

ANOVAs for response amplitude and time to peak did not reveal significant interactions between genotype and brain region but discovered an overall genotype effect: the evoked BOLD signal amplitude was significantly higher in HCN4-KO compared to Ctr, again most evident at 45°C (Figure 3B). In addition, a longer time to peak was observed in HCN4-KO (Figure 3C). These results indicate a change in the overall dynamics of nociceptive processing. Interestingly, low temperatures resulted in a faster time to peak and low amplitudes, whereas high temperatures evoked high amplitudes that also took longer to reach (Supplementary Figure S3).

Finally, the percentage activation probability across all brain regions was also higher for HCN4-KO at all four stimulus temperatures (Figure 3D) but did not reach significance threshold.

As these results showed highly significant differences between HCN4-KO and Ctr at the stimulation temperature of 45°C, we next focused on this temperature as well as the next higher temperature of 50°C. This was not chosen arbitrarily, as we know that the threshold between warmth and heat detection in rodent thermal fMRI lies between these two temperatures (Wank et al., 2022). This allowed us to obtain information about thermal discrimination and nociceptive processing simultaneously without having to account for endogenous descending inhibition, which is thought to be active at around 54°C.

Since the previous results suggest differences in information processing, graph-theoretical analyses based on functional connectivity were applied. This allowed deep insight into brain network properties: connection strength between brain regions (= nodes), clustering coefficient (high values indicate informational segregation but also local integration), shortest pathlength (least number of steps needed to connect two brain regions; short paths lead to fast processing, indicating high integration, whereas long paths indicate higher segregation), hubscore and betweenness (two measures to estimate the importance of nodes for network integrity and information flow). As a ratio of normalized clustering coefficient and normalized shortest pathlength, the small world index sigma represents the overall information processing efficacy of the brain network.

**FIGURE 4:**
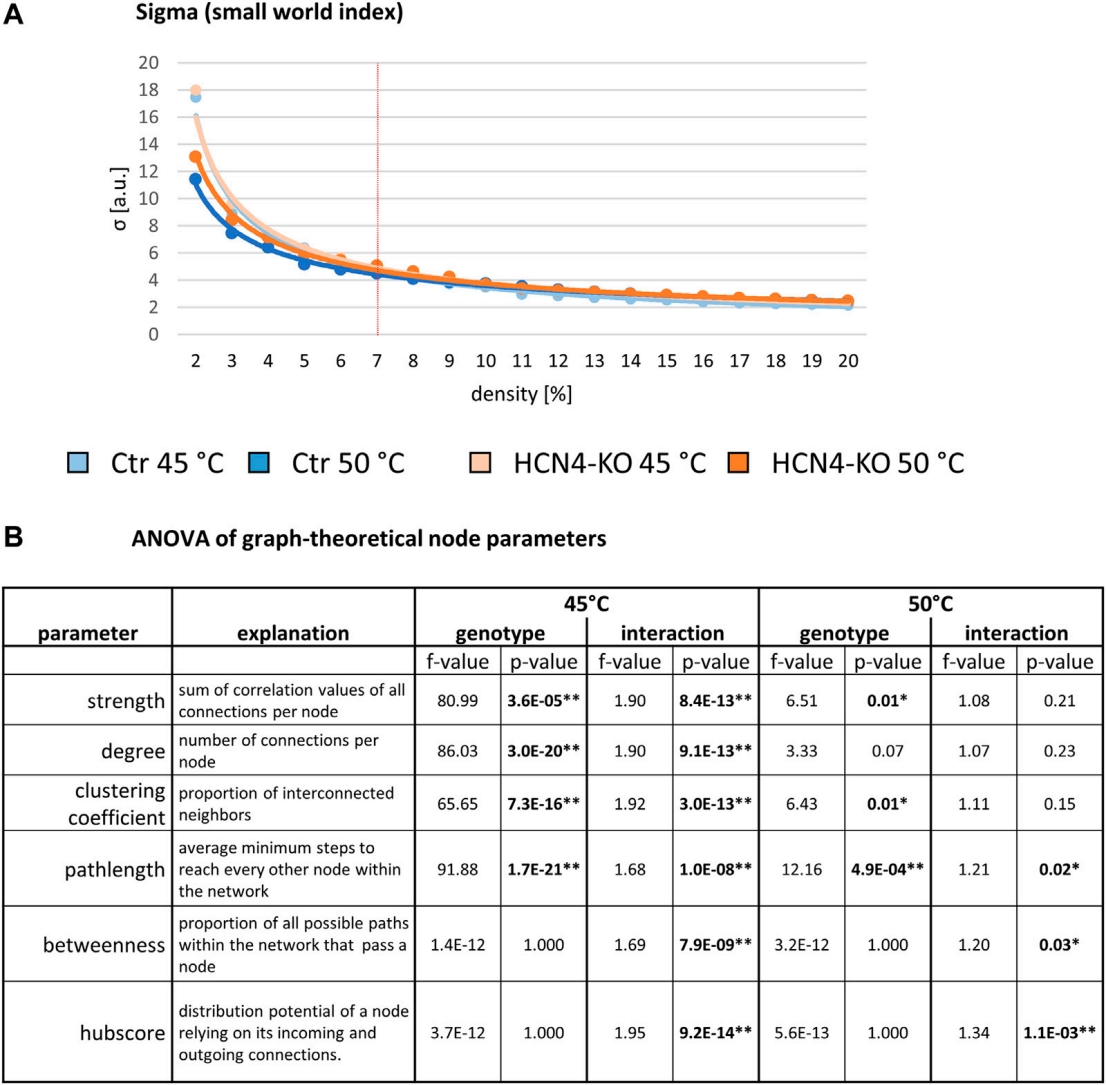
Global graph-theoretical parameters for stimulus-driven BOLD fMRI. **(A)** Whole-network parameters for 45°C and 50°C: Small World Index sigma plotted for network densities ranging from 2% to 20%. The red vertical line demarks the determined 7% threshold with which all further analyses were performed. **(B)** Node-specific parameters for 45°C and 50°C: Two-factor ANOVA for node parameters revealed significant differences between Ctr and HCN4-KO in general, but also showed interactions between the factors genotype and brain region, indicating region-specific modulation through HCN4 channels. *p < 0.05, **p < 0.01 were determined by a two-factor ANOVA with one within-factor containing the 211 brain regions and one between-factor genotype (Ctr, *n* = 10 and HCN4-KO, *n* = 10). Significant differences were found at 45°C for strength, degree, clustering coefficient, pathlength, betweenness and hubscore, for 50°C only for the latter three parameters.

As these measures are highly dependent on network size (density), we first determined the 7% network density at which the most pronounced change in efficacy occurred (see Methods). Next, sigma was calculated for 45°C and 50°C networks of Ctr and HCN4-KO, starting at a density of 2%, increasing in 2%-steps up to 20%: sigma showed a decrease with increasing density in all four data sets. At a density of 7%, Ctr and HCN4-KO were not significantly different at either 45°C or 50°C (Figure 4A).

Analysis of the graph-theoretical node parameters by two-factor ANOVA revealed significant main effects between Ctr and HCN4-KO at 45°C for all node parameters except betweenness and hubscore (normalized values). Interactions between genotype and brain region were found for all these node parameters, suggesting that these main effects are driven by specific brain regions (Figure 4B). At 50°C, significant main effects between Ctr and HCN4-KO were found for the parameters strength, clustering coefficient, and pathlength, as well as interactions for the parameters pathlength, betweenness, and hubscore.

After identifying the differing brain regions using a homoscedastic *t*-test as *post hoc* test, we found that (Table 3):

1) Strength and degree were significantly increased at 45°C for HCN4-KO in structures of the amygdala as well as in parts of the cerebellum, hypothalamus, and sensory input, indicating a better-connected network of HCN4-KO in the aforementioned structures.
2) The clustering coefficient was significantly increased in HCN4-KO at 45°C in structures of the amygdala, cerebellum, hypothalamus, and sensory input, but also in parts of the sensory cortex and the basal ganglia, indicating higher segregation in HCN4-KO.
3) The pathlength was significantly longer at 45°C for HCN4-KO in structures of the amygdala, hypothalamus, sensory input, and sensory cortex (45°C: higher segregation in HCN4-KO). At 50°C, the percentage difference for HCN4-KO was significantly higher in parts of the thalamus, association cortex and amygdala, while it was lower in structures of the brainstem, sensory input, link to the limbic system (piriform and rhinal cortex), hippocampus, and cerebellum (50°C: mixed findings for HCN4-KO).
4) The betweenness was significantly higher at 45°C for HCN4-KO compared to Ctr in structures of the amygdala, hypothalamus, olfactory, and sensory input (45°C: higher importance in HCN4-KO). At 50°C, the percentage difference showed higher values in HCN4-KO especially in parts of the association cortex, but also in parts of the limbic system, amygdala, and basal ganglia. Lower values for HCN4-KO were found in structures of the brainstem, sensory input, sensory cortex, and cerebellum (50°C: mixed findings for HCN4-KO).
5) The hubscore at 45°C was only slightly higher for HCN4-KO in a substructure of the cerebellum (45°C: almost no difference between HCN4-KO and Ctr). At 50°C, HCN4-KO showed higher values in the link to the limbic system and in structures of the amygdala, but lower values in thalamic structures and in the hippocampus (50°C: slightly higher importance in HCN4-KO).

**TABLE 3:** Brain region-specific graph-theoretical parameters for stimulus-driven BOLD fMRI.

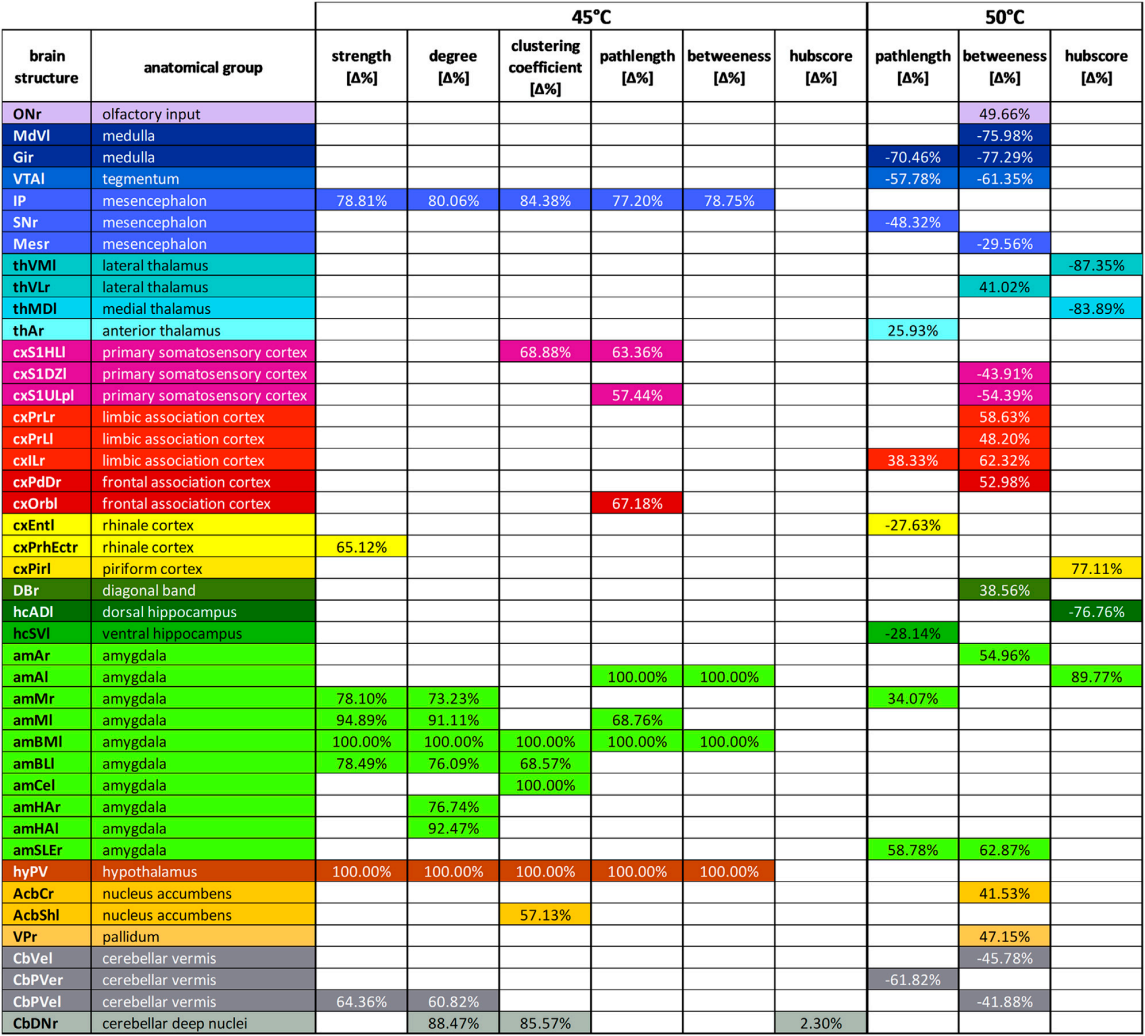

Significant differences in node parameters expressed as percentage change from Ctr to HNC4-KO (positive percentages indicate an increase in HCN4-KO). A homoscedastic *t*-test was used as *post hoc* test for brain regions (*p* < 0.05; uncorrected). All parameters were tested for both temperatures. Only node parameters that showed significant differences between HCN4-KO and Ctr and within those only significantly different brain regions are shown. For 45°C in particular, the amygdala (light green) showed higher values for HCN4-KO in several parameters. For 50°C, mainly brain stem (blue) and cortical (pink) regions showed differences in the parameter betweenness. Abbreviations: l, left; r, right; ONr, olfactory nuclei; MdVl, ventral medullary reticular nucleus; Gir, gigantocellular reticular nucleus; VTAl, ventral tegmental area; IP, interpeduncular nucleus; SNr, substantia nigra; Mesr, mesencephalic region; thVMl, ventromedial thalamic nucleus; thVlr, ventrolateral thalamic nucleus; thMDl, mediodorsal thalamus; thAr, anterior thalamic group; cxS1HLl, primary somatosensory cortex hind limb; cxS1DZl, primary somatosensory cortex dysgranular; cxS1ULpl, primary somatosensory cortex upper lip; cxPrLr, cxPrLl, prelimbic cortex right and left; cxlLr, infralimbic cortex; cxPdDr, dorsal peduncular cortex; cxOrbl, orbital cortex; cxEntl, entorhinal cortex; cxPrhEctr, perirhinal/ectorhinal cortex; cxPirl, piriform cortex; DBr, nuclei of diagonal band; hcADl, anterior dorsal hippocampus; hcSVl, ventral subiculum; amAr, amAl, anterior amygdala right and left; amMr, amMl, medial amygdaloid nucleus right and left; amBMl, basomedial amygdaloid nucleus; amBLl, basolateral amygdaloid nucleus; amCel, central nucleus of the amygdala; amHAr, amHAl, amygdala hip area right and left; amSLEr, sublenticular extended amygdala; hyPV, periventricular hypothalamic nucleus; AcbCr, core subregion of the nucleus accumbens; AcbShl, shell subregion of the nucleus accumbens; VPr, ventral pallidum; CbVel, cerebellar vermis; CbPVer, CbPVel, cerebellar paravermis right and left; CbDNr, cerebellar deep nuclei.

In addition, changes in the flow of information between individual brain regions can be analyzed: Using network-based statistics, differences in functional connectivity (FC) between Ctr and HCN4-KO were calculated and then displayed as 3D networks. Although the differences found in FC did not reach the significance level at either 45°C or 50°C stimulus, the analysis indicated that HCN4-KO tended to have a higher FC compared to Ctr (Supplementary Figure S4A). At 45°C, FC in HCN4-KO was higher in structures of the brainstem, sensory input (tegmentum), thalamus, sensory cortex, olfactory regions, limbic system, in the amygdala especially at the left side, the hypothalamus, and the cerebellum. In Ctr group, FC was higher in some structures of the sensory cortex and the basal ganglia.

The difference in FC at 50°C is shown in (Supplementary Figure S4B). For HCN4-KO, the FC was higher in structures of the sensory input (colliculi), thalamus, association cortex, olfactory regions, link to the limbic system, limbic system, amygdala, hypothalamus, and cerebellum. Again, for Ctr, FC was higher only in a few structures, most notably the hippocampus.

### 3.3 Resting state analysis

**FIGURE 5:**
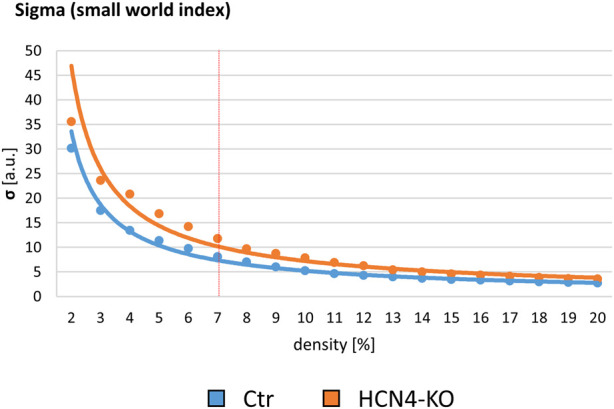
Baseline resting state graph-theoretical assessment. Small World Index sigma plotted for network densities ranging from 2% to 20%. The red vertical line demarks the determined 7% threshold with which all further analyses were performed. In order to assess any genotype-specific differences in baseline brain functional connectivity at rest, pre RS Ctr was compared to pre RS HCN4-KO. HCN4-KO here showed slightly higher sigma values.

As shown for the s-fMRI data, RS sigma curves of Ctr and HCN4-KO showed a decrease over the density. Overall, Ctr had lower sigma than HCN4-KO. At 7% density, Ctr and HCN4-KO were not significantly different (Figure 5). To assess overall differences in RS brain functionality, two-factor ANOVAs between Ctr and HCN4-KO with the factors genotype and brain region were calculated for all node-specific parameters of the pre RS networks, as described above for stimulus-driven data. In contrast to the stimulus-driven data, there was no interaction between genotype and brain region for pre RS, indicating that the overall efficacy of the RS brain networks was unaffected by HCN4-KO.

**FIGURE 6:**
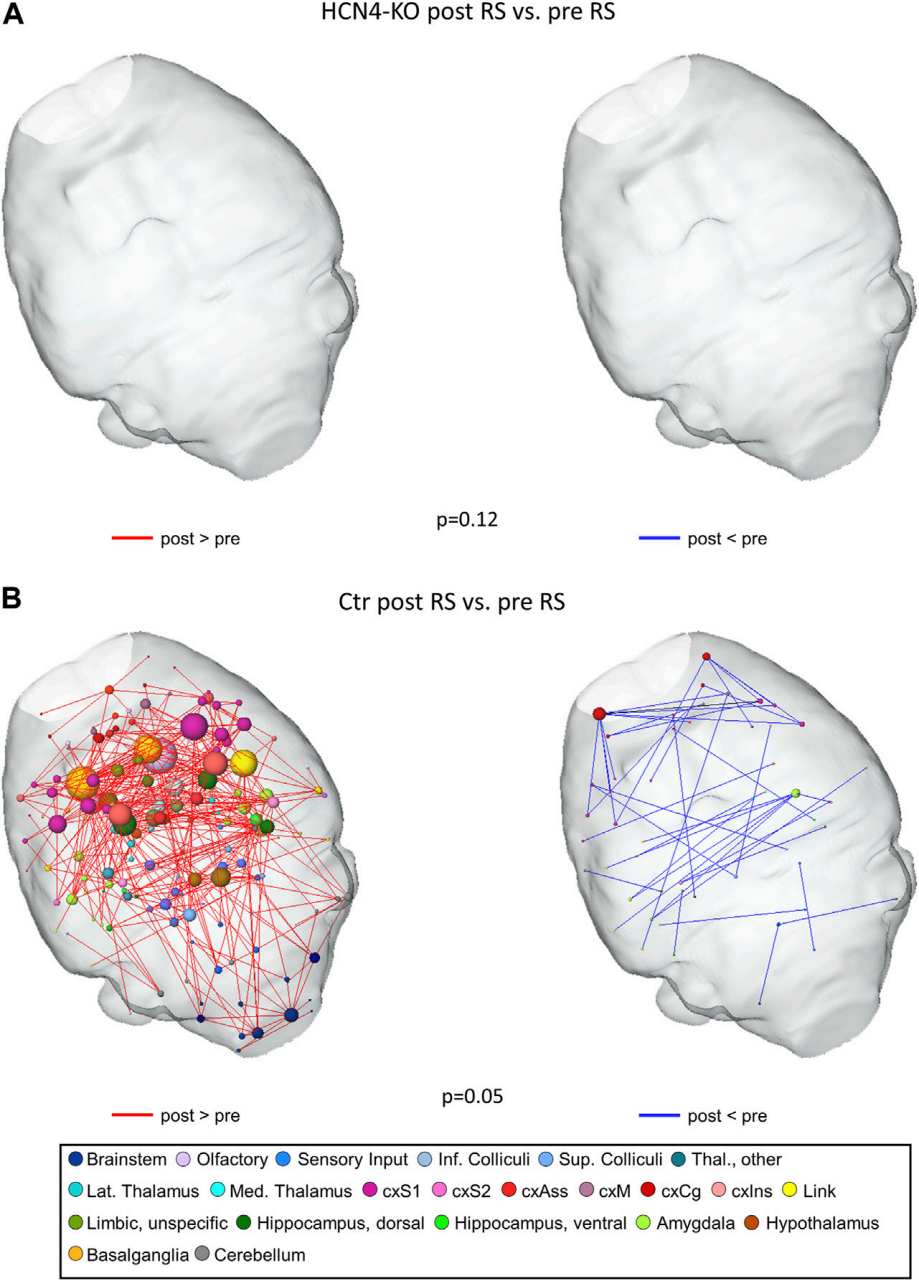
Thermal stimulation significantly modulated resting state functional connectivity in Ctr but not HCN4-KO. For that purpose, we compared pre RS to post RS for either Ctr (*n* = 10) or HCN4-KO (*n* = 10). Differences were calculated using network-based statistics, (*p*-values as indicated) and displayed as 3D-networks within a transparent brain surface. Color-coded nodes represent one out of 206 single brain regions (orphan nodes are not shown). Edges between the nodes represent significant differences in RS functional connectivity (red: post RS > pre RS; blue post RS < pre RS) evoked by the intermittent thermal stimulation period. HCN4-KO did not show significant differences overall (*p* = 0.12), therefore the brains are shown blank **(A)**. In contrast, the thermal stimulation significantly modulated RS functional connectivity in Ctr mice **(B)** in brainstem (blue nodes), amygdala (light green), basal ganglia (darker yellow), olfactory tubercle (lilac), hippocampal (dark green) and cortical (pink) areas. Abbreviations: cxS1, primary somatosensory cortex; cxS2, secondary somatosensory cortex; cxAss, association cortex; cxM, motor cortex; cxCg, cingulate cortex; cxlns, insular cortex.

It is well known that even transient and non-salient stimuli can leave lasting imprints on RS brain networks (Kreitz et al., 2018). By comparing RS networks from before to after thermal stimulation, modulatory effects of the stimulation can be detected: Analyzing the RS FC between pre and post RS measurement using network-based statistics, overall FC was higher in post RS than in pre RS for both groups. No significant differences were found for HCN4-KO (Figure 6A). For the Ctr group, differences in FC were significantly higher in post RS than in pre RS, especially in areas of the brainstem, sensory input, thalamus, sensory cortex, olfactory regions, link to the limbic system, limbic system, and hippocampus (Figure 6B).

### 3.4 Analysis of anatomical data

After completion of the functional measurements, *ex vivo* high-resolution anatomical MRI was performed: Both voxel-based and deformation-based morphometry were used to assess total brain volume and local regional differences.

**FIGURE 7:**
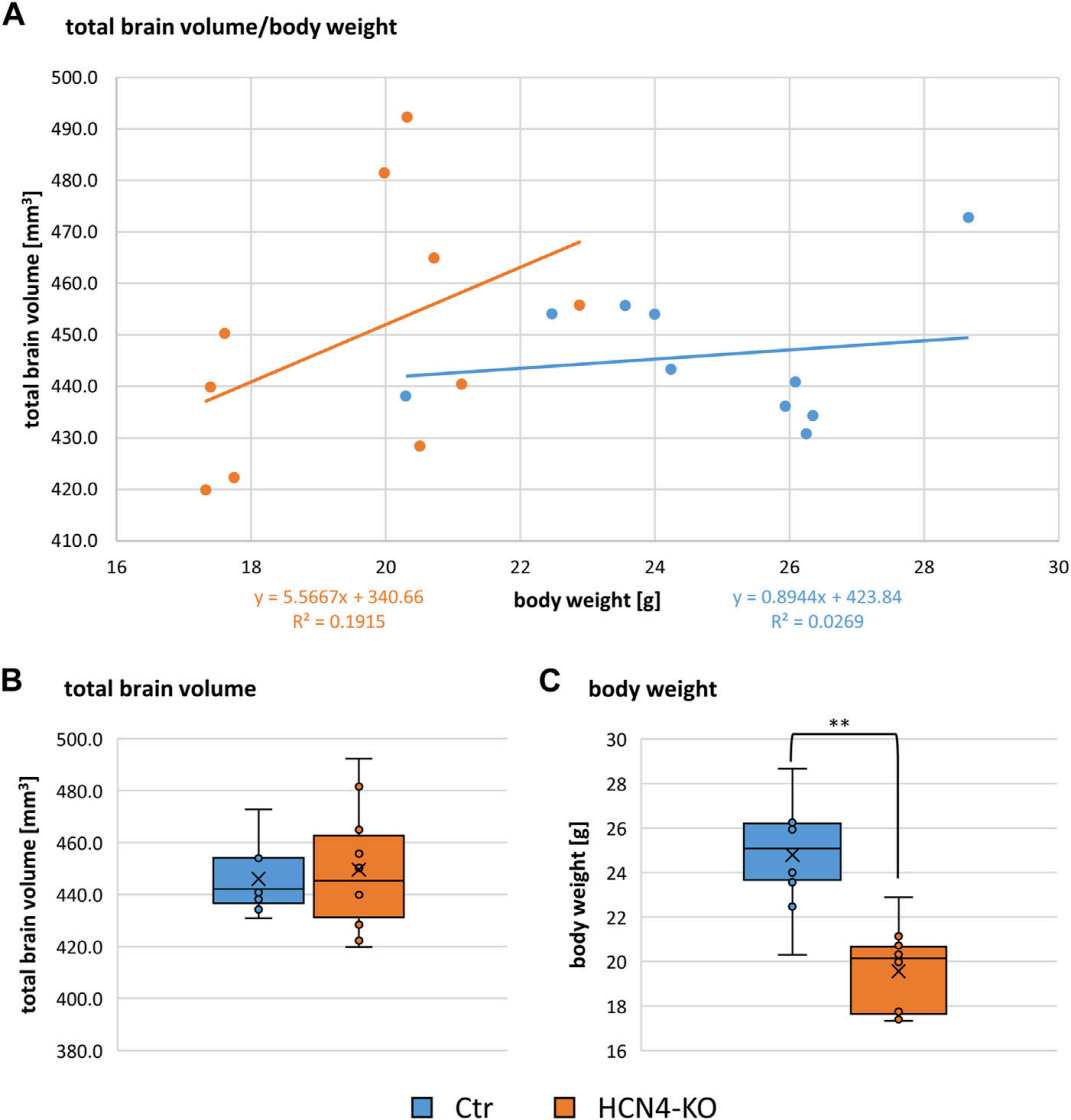
Relation of brain volume to body weight. **(A)** Dot plot of total brain volume in relation to the body weight for Ctr (*n* = 10) and HCN4-KO (*n* = 10). Box plots of total brain volume **(B)** and body weight **(C)** compare Ctr and HCN4-KO. Box spans the 25th to the 75th percentiles, the median is represented by the solid horizontal line and the mean is marked with the x. The individual values from all animals are displayed as dots. Ctr (*n* = 10) and HCN4-KO (*n* = 10); *p < 0.05, **p < 0.01 were determined by a one-factor ANOVA. The HCN4-KO mice had significantly lower body weight but interestingly no difference in their brain volume.

**FIGURE 8:**
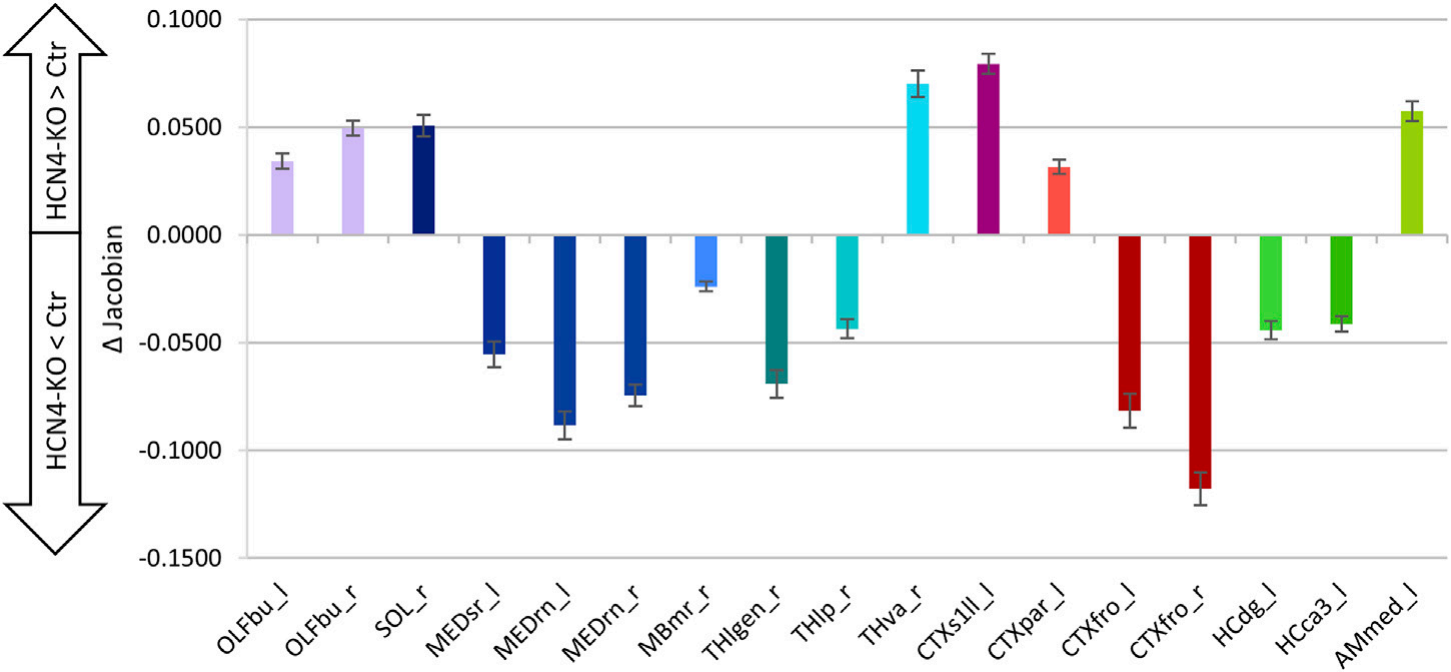
Brain region-specific volume differences. Shown are differences (HCN4-KO subtracted from Ctr) between mean Jacobian determinants ±SE of the difference (homoscedastic Student’s *t*-test, uncorrected). Local volume differences can be assessed by comparing the deformation fields expressed as Jacobian determinants. These values represent the amount of the deformation vector for voxel displacement, but not the direction. Positive differences indicate enlarged regional volume for HCN4-KO, while negative differences indicate reduced regional volume for HCN4-KO. Ctr (*n* = 10) and HCN4-KO (*n* = 10). Compared to Crl, HCN4-KO showed larger olfactory bulb (OLFbu_l, OLFbu_r), left primary somatosensory cortex (CTXs1ll_l) and left amygdala (AMmed_l). Volume of medulla (MEDsr_l, MEDrn_l, MEDrn_r), frontal cortical (CTXfro_l, CTXfro_r) and hippocampal regions (HCdg_l, HCca3_l) were reduced in HCN4-KO. Abbreviations: l, left; r, right; OLFbu_l, OLFbu_r, olfactory bulb left and right; SOL_r, nucleus of the solitary tract; MEDsr_l, medulla sensory related; MEDrn_l, MEDrn_r, medulla raphe nuclei left and right; MBmr_r, midbrain motor related; Thlgen_r, lateral geniculate complex; THlp_r, lateral posterior nucleus of the thalamus; Thva_r, ventral anterior-lateral complex of the thalamus; CTXs1ll_l, primary somatosensory area lower limb; CTXpar_l, posterior parietal association areas; CTXfro_r, frontal pole; HCdg_l, dentate gyrus; HCca3_l, field CA3; AMmed_l, medial amygdalar nucleus.

The relationship between total brain volume and body weight is summarized in (Figure 7A). There were no significant differences in total brain volume (Figure 7B), although brain-specific knockout of HCN4 resulted in significantly lower body weight (Figure 7C). For a deeper insight into individual brain regions, local volume differences were analyzed using the deformation-based Jacobian determinants (Figure 8). Positive values indicate a volume increase of the brain structure for HCN4-KO compared to Ctr, while negative values denote a decrease in size. This analysis indicates that mainly structures of the olfactory system, the ventral anterior-lateral complex of the thalamus, primary somatosensory areas, posterior parietal association areas as well as parts of the amygdala were larger in HCN4-KO than in Ctr. On the other hand, brainstem structures (medulla), parts of the thalamus, motor-related parts of the midbrain, parts of the association cortex, as well as parts of the hippocampus were smaller in HCN4-KO.

## 4 Discussion

Previous studies found that HCN channels play a role in nociceptive processing, especially in the context of inflammatory and neuropathic pain. Experiments using the non-selective HCN channel blocker ZD7288 were able to significantly reduce chronic pain, but unfortunately did not allow conclusions to be drawn about the channel types involved (Luo et al., 2007).

A comparison of HCN2 channels—already well-studied in the aspect of pain behavior—with HCN4 channels shows that both HCN isoforms are very similar in their basic molecular characteristics (cation channels activated by hyperpolarized membrane potential and modulated via cAMP) (Wahl-Schott and Biel, 2009), but differ in their activation kinetics (Ludwig et al., 1999; Seifert et al., 1999).

Both channels differ in their expression pattern (Santoro et al., 2000; Heintz, 2004). While HCN2 channels are mostly ubiquitously expressed in the brain (Moosmang et al., 1999), HCN4 channels are mainly expressed in the thalamus (Moosmang et al., 1999), a region known to be essential for central information processing (Herrero et al., 2002), but also in structures of the olfactory system (Fried et al., 2010; Nakashima et al., 2013), hippocampus (Hughes et al., 2013), the cerebellum (Zuniga et al., 2016), and the basolateral amygdala (Monteggia et al., 2000).

These aspects suggest that HCN4 may also play a similar role in nociception and may turn out to be a future target for pain therapy. Therefore, we aimed to improve our knowledge of HCN4 by analyzing a brain-specific knockout mouse model with respect to its peripheral and central nociception in general and the importance of the HCN4 channel for nociceptive processing and brain functionality and anatomy in particular.

Interestingly, the HCN4-KO has been shown to be reflected in thermal hypersensitivity and increased anxiety behavior, contrary to initial expectations based on the findings with HCN2-KO models (Schnorr et al., 2014). Our findings primarily suggests a centrally mediated thermal hypersensitivity, since the tail flick as a spinal peripheral reflex (Irwin et al., 1951) remained unaffected, whereas the stimuli of the Hargreaves test are processed more centrally (Hargreaves et al., 1988). In contrast, HCN2 channels are mainly involved in mechanosensation, as the mechanical perception is reduced in HCN2-knockout mice (Schnorr et al., 2014). However, it is difficult to make a comparison because most publications using HCN2-KO models have only investigated peripheral effects. For better comparisons in the future, the effects of HCN2-KO on the CNS need to be investigated further using a comparable experimental design.

As described above, fMRI data can be analyzed from different aspects: conventional BOLD signal analysis focuses on the quantification of the BOLD response, whereas graph-theoretical analyses provide insight into the information processing within the whole brain network.

Since a conventional BOLD signal analysis does not assess the mutual influence of brain structures, the graph-theoretical analysis focused on exactly these interactions, i.e., the FC between brain regions: on the one hand by node-specific parameters and on the other hand by whole network interactions.

Notably, HCN4-KO showed a significantly longer time to peak (Figure 3C), indicating a different timing of connectivity of brain structures compared to Ctr.

The node parameters can be used to determine, among other things, how strongly the individual brain regions within the network are connected to each other. These parameters can also be used to determine how important a particular brain structure is for the functioning of a network and whether information processing is distributed throughout the whole network or concentrated in certain nodes or regions.

Integrating the results of functional and anatomical analyses, we found profound alterations in structures of the sensory input, the primary somatosensory cortex, the cerebellum, the amygdala, and the hypothalamus. As already mentioned, HCN4 channels have been shown to be expressed in these structures. In the HCN4-KO, all of them showed a significantly increased activated volume, an increased participation in the processing of the thermal stimuli—the node parameters indicated a more efficiently connected but decentralized network with respect to the structures mentioned—and some also had a partially altered volume regarding the anatomical size of the brain region.

Interestingly, these differences in the activated brain volume and their involvement in the information processing of the above-mentioned structures between HCN4-KO and Ctr were observed especially at the warm temperature of 45°C, but not at noxious temperatures (50°C and 54°C). This finding supports the hypothesis of a central thermal hypersensitivity in HCN4-KO, which was also found in the behavioral Hargreaves test.

Graph-theoretical evaluation of the data revealed differences in areas of the somatosensory cortex as well as the hypothalamus and the cerebellum. Examination of the anatomical results even revealed that parts of the somatosensory cortex were larger in HCN4-KO than in Ctr. In addition to all kinds of tactile stimuli such as pressure or vibration, the somatosensory cortex also processes thermal stimuli (Burton and Sinclair, 1996) and thus plays a role in the perception of pain (Bushnell et al., 1999). The increased activity in the somatosensory cortex in HCN4-KO, especially at warm temperatures (45°C), as well as its partially enlarged brain region volume indicate an increased perception of thermal stimuli. Interestingly, at the noxious temperature (50°C), the importance of the somatosensory cortex for the processing of thermal stimuli decreased, which is consistent with its function in the lateral pain pathway to localize pain (Treede et al., 1999).

In the same context, cerebellar structures are also known to be involved in the processing of painful stimuli, although this is not fully understood. However, fMRI studies have shown that cerebellar structures are activated under painful conditions (Borsook et al., 2008; Tseng et al., 2010; Moulton et al., 2011). Cortical input travels via the brainstem to the cerebellum and from there to the thalamus and hypothalamus (Moulton et al., 2010).

Thus, the cerebellum has a kind of gating function. In our case, both the increased activity in the stimulus response analysis and the observation of higher functional connectivity in HCN4-KO during the application of weaker thermal stimulation (45°C) indicate an important role of the cerebellum in nociceptive processing. However, as with the somatosensory cortex, brainstem, and sensory input, information processing decreased with higher thermal stimulation (50°C). At 50°C, it can be observed that the influence of the above-mentioned structures is now stronger in the Ctr group. This could indicate that these structures, including the cerebellum, are involved in the ability to discriminate between innocuous and noxious stimuli.

A possible explanation for the increased anxious behavior of HCN4-KO mice in the open field test could be the influence of the amygdala, which, according to the analysis of the node-specific parameters, had a significant impact on information processing in HCN4-KO, especially at 45°C but also at 50°C. This is also supported by an increased amygdala volume in HCN4-KO compared to Ctr.

As shown previously, the HCN4 channel is indeed expressed in the amygdala (Monteggia et al., 2000; Santoro et al., 2000; Park et al., 2011). *In vitro* data support an important role for HCN channels in controlling the overall excitability of the basolateral amygdala by enhancing the inhibitory control. A blockade of the HCN channels in the basolateral amygdala leads to an increase in basolateral amygdala excitability, causing anxiety-like behavior (Park et al., 2011). Interestingly, a dorsal hippocampus-specific knockdown of the HCN4 channel has also been reported to increase anxiety-like behavior in mice (Gunther et al., 2019). While our results indicate that the amygdala is an important structure for information processing in HCN4-KO, hippocampal structures were found to be less important.

The hypothalamus also plays an important role in the processing of stressful events. In animal models, an increased release of norepinephrine has been observed in hypothalamic structures induced by stress, which may be related to the induction and development of anxious behavior (Tanaka et al., 2000). The pronounced activity of the hypothalamus and its importance for brain network efficacy in our case could thus also indicate an increased sensitivity to weaker thermal stimuli and a general increase in anxious behavior. Taken together, this could explain the higher anxiety score found in the open field test.

As transient thermal stimulation is known to modulate RS FC (Kreitz et al., 2018; Wank et al., 2022), we also performed RS measurements directly before and after the stimulus-driven fMRI measurement to investigate the short-term modulatory influence of thermal stimuli on basic resting brain function. Since no significant differences were found between HCN4-KO and Ctr in the pre RS measurement, this leads to the conclusion that the brain-specific knockout of HCN4 does not affect basic resting brain function in general.

Comparing post and pre RS measurements within each group, HCN4-KO and Ctr, showed that only in Ctr a significant difference between post and pre RS was found. Such short-term modulatory effects as seen in RS in the Ctr group are usually observed after preceding painful experiences and are known to initialize aversive memory formation in the hippocampus (McEwen, 2001).

A previous study showed that lesion of the hippocampus resulted in decreased passive avoidance of painful events in rats (Blanchard and Fial, 1968). It was also shown that a lesion of the ventral hippocampus shortly after birth resulted in decreased latency in thermal pain-relevant behavioral tests in juvenile but not adult rats (Al Amin et al., 2004). While neuroplasticity can rescue the lesion effects as the animal ages, a gene knockout, as used in our experiments, is irreversible. Therefore, the fact that HCN4-KO mice do not display Ctrl-typical changes in hippocampal FC (during s-fMRI at 50°C and RS) could lead to the conclusion that HCN4-KO mice may not be able to remember noxious experiences in the same way as mice from the Ctr group. Further studies over a longer period might demonstrate sustained modulatory effects on nociceptive memory formation.

The fact that functional changes were also accompanied by changes in brain anatomy (especially with respect to genotype-specific brain anatomy) underscores the importance of correctly adapting the brain atlas to the individual anatomy for fMRI analyses, even if this requires special adaptations (e.g., adding, skipping or exchanging slices). However, the significantly lower body weight associated with HCN4-KO was not reflected in a reduced total brain volume as shown by the anatomical data, and therefore does not seem to have an influence on information processing.

Regarding the limitations of our study, we had a limited number of animals with the desired genotype due to reduced breeding rates.

We also present uncorrected *p*-values in some cases: the ANOVA revealed highly significant interactions between genotype and brain region, but the structure-wise *post hoc* test yielded only *p*-values close to the significance threshold of *p* < 0.05, which did not survive FDR correction for multiple comparisons. We still present these values, clearly marked, as they often indicate important trends and are valuable for discussion.

A general confounding factor is anesthesia during the fMRI measurement. Isoflurane was used as anesthetic to minimize the effect of anesthesia on brain function. Despite known dose-dependent effects of isoflurane anesthesia on cerebral blood flow and glucose metabolism in humans, as well as its influence on neurotransmitter signaling (Lenz et al., 1998; Masamoto et al., 2009), low-dose isoflurane allows for an easily adjustable anesthesia that is only mildly analgesic, an important fact considering the research topic of nociception. Compared to other narcotics, it shows less influence on brain activity in general. This influence is expressed in a quantitative rather than a qualitative difference (Hess et al., 2007), resulting in a slightly attenuated activation amplitude but activation patterns comparable to the awake state (Chang et al., 2016). To draw reliable conclusions from the experiments, it is necessary that all experimental animals are treated with the same anesthetic regime during the MRI measurement. No significant differences in isoflurane dose were required between animals to achieve a stable respiratory rate, which is of paramount importance for the BOLD signal. Because human fMRI is usually performed in the awake state, results from anesthetized animals may differ in some aspects. Nevertheless, previous studies have shown that results from nociceptive and RS-fMRI are generally highly translational (Hess et al., 2011; Sierakowiak et al., 2015).

Moreover, we would like to point out that a further distinction of the different somatosensory pathways in the thalamus into epicritic (tractus spinobulbaris) and protopathic (tractus spinothalamicus anterior and lateralis) is functionally difficult given the many collaterals between the two tracts. Due to both slow temporal and limited spatial resolution, it is currently not possible to separate these two pathways in MRI data.

In summary, based on the behavioral tests, HCN4-KO did not impair locomotion, but resulted in a hypersensitive phenotype with respect to thermal perception. Analysis of s-fMRI, RS and anatomy identified the hypothalamus, somatosensory cortex, cerebellum, and especially the amygdala as key regions.

## Data Availability

The raw data supporting the conclusion of this article will be made available by the authors, without undue reservation.
