# Supplementary figures and images for: The impact of HCN4 channels on CNS brain networks as a new target in pain development
Source: Front Netw Physiol. 2023 Jul 10;3:1090502. doi: 10.3389/fnetp.2023.1090502 (PMC10368246; doi:10.3389/fnetp.2023.1090502)

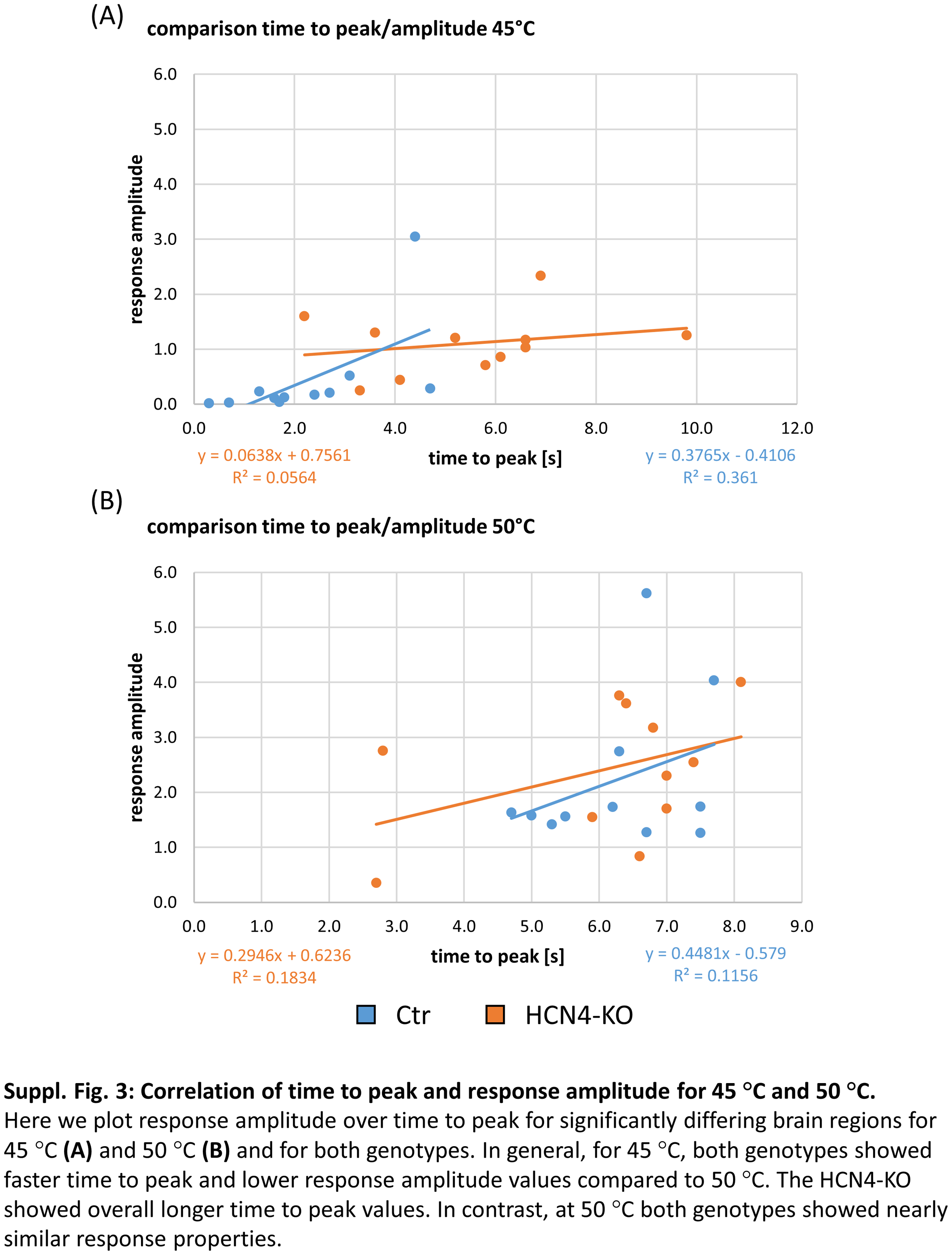

Supplement: Supplementary file 1 [file Image3.TIF]

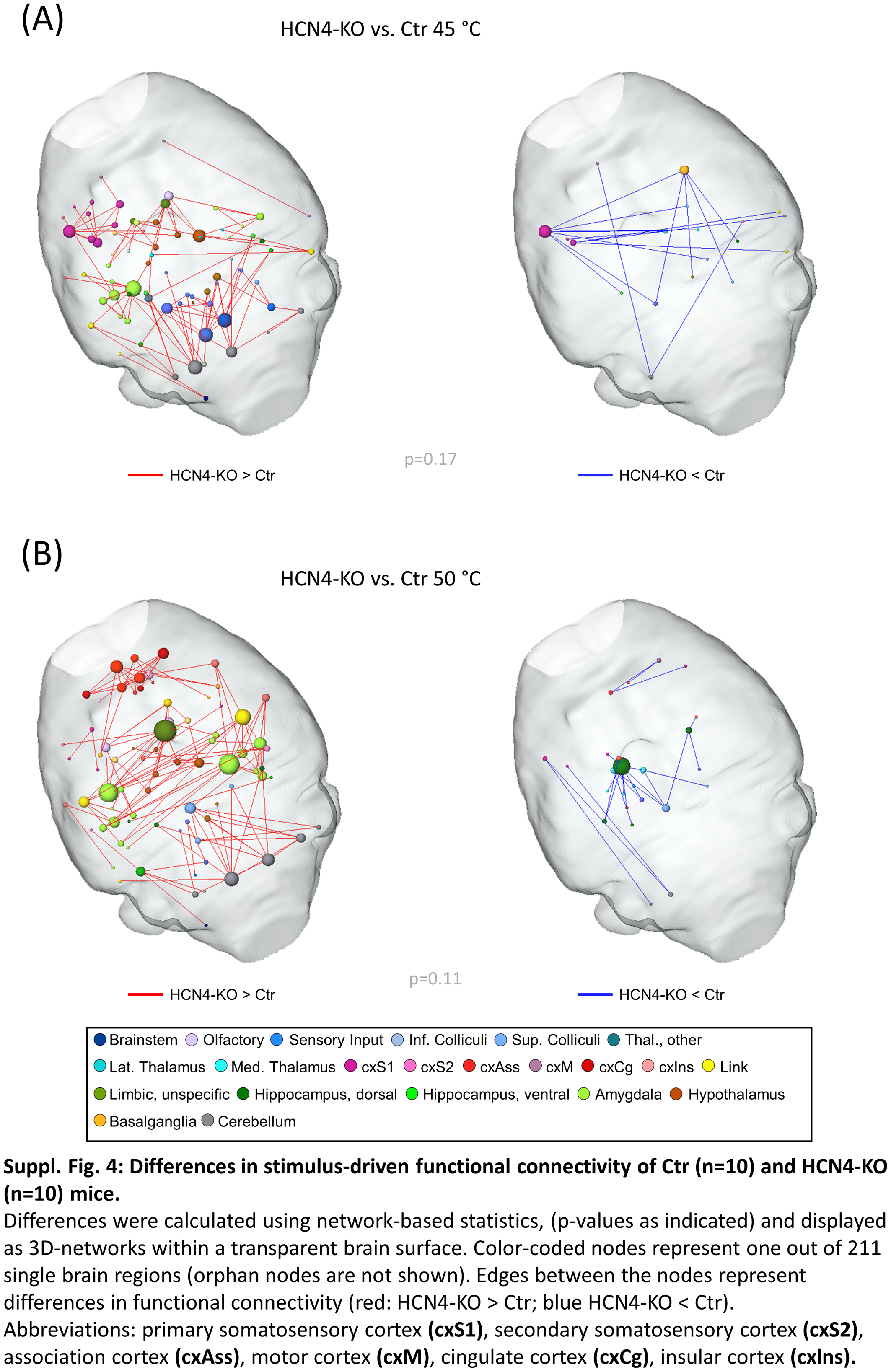

Supplement: Supplementary file 2 [file Image4.tif]

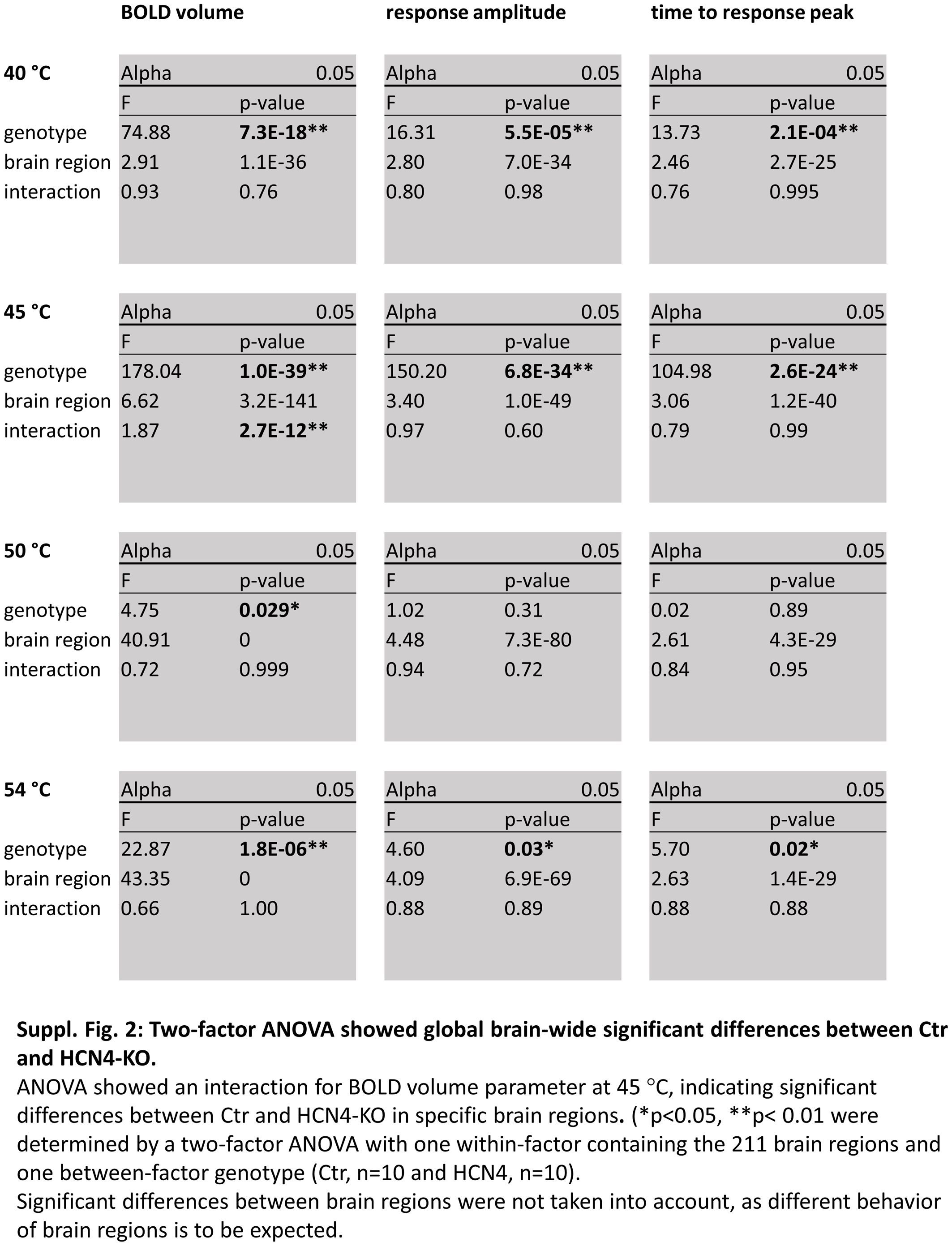

Supplement: Supplementary file 3 [file Image2.TIF]

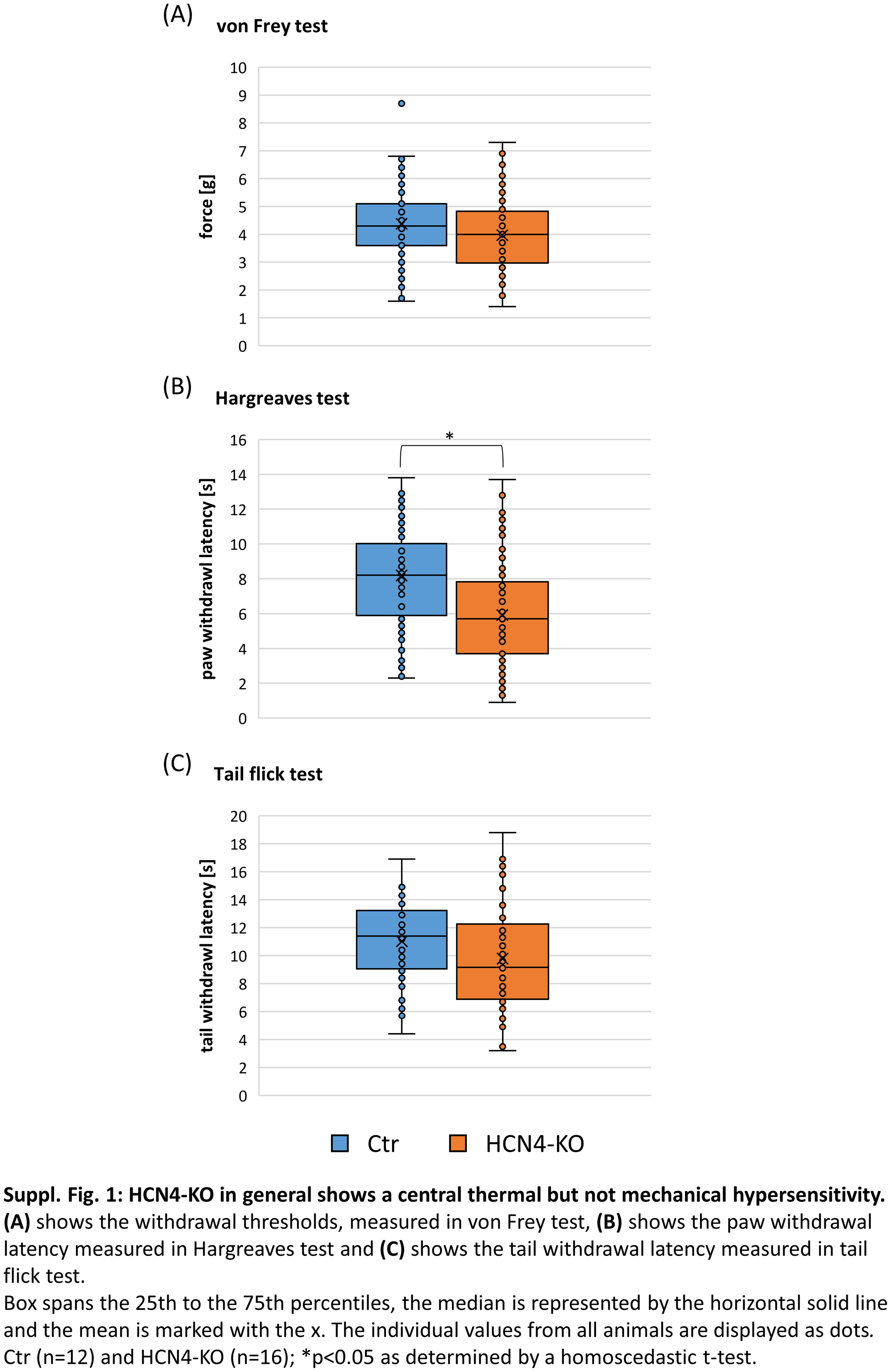

Supplement: Supplementary file 4 [file Image1.TIF]
